# RNA-directed peptide synthesis across a nicked loop

**DOI:** 10.1093/nar/gkae702

**Published:** 2024-08-21

**Authors:** Meng Su, Samuel J Roberts, John D Sutherland

**Affiliations:** MRC Laboratory of Molecular Biology, Francis Crick Avenue, Cambridge Biomedical Campus, Cambridge CB2 0QH, UK; MRC Laboratory of Molecular Biology, Francis Crick Avenue, Cambridge Biomedical Campus, Cambridge CB2 0QH, UK; MRC Laboratory of Molecular Biology, Francis Crick Avenue, Cambridge Biomedical Campus, Cambridge CB2 0QH, UK

## Abstract

Ribosomal translation at the origin of life requires controlled aminoacylation to produce mono-aminoacyl esters of tRNAs. Herein, we show that transient annealing of short RNA oligo:amino acid mixed anhydrides to an acceptor strand enables the sequential transfer of aminoacyl residues to the diol of an overhang, first forming aminoacyl esters then peptidyl esters. Using *N*-protected aminoacyl esters prevents unwanted peptidyl ester formation in this manner. However, *N*-acyl-aminoacyl transfer is not stereoselective.

## Graphical Abstract

**Graphical Abstract:**
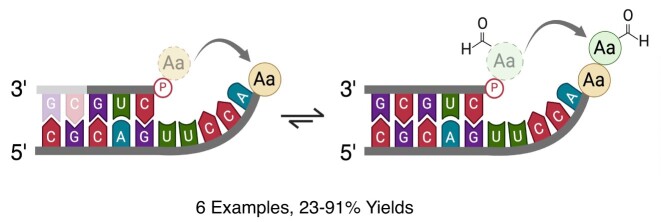

## Introduction

Modern RNA-coded peptide synthesis is complicated, including the ribosome and various translation factors to enable peptidyl transfer at the (*N*-acyl-)aminoacylated 3′-CCA termini of two juxtaposed tRNAs (1). Although the details of this mechanism have been elucidated in recent years (2,3), it is not known how translation could have first emerged.

According to the RNA world hypothesis (4), first posited in 1986, self-replicating RNA molecules proliferated before the evolution of DNA and proteins. In support of this proposal, RNA has been found to be capable of both storing genetic information and catalysing essential biochemical reactions. Given RNA’s key role in the synthesis of proteins there has been a long-standing interest in understanding how translation developed on early Earth. Several cases of RNA-catalysed or RNA-mediated peptide synthesis have been reported. A 196-nt ribozyme was evolved to perform a peptidyl transfer reaction to yield Met-Phe dipeptide (5,6). A triphenylalanyl-RNA ester was formed in a nicked RNA/DNA duplex without additional ribozymes (7). Oligophenylalanine up to a pentapeptide was detected using a five-nucleotide ribozyme and phenylalanyl adenylate (8). However, whether phenylalanine can be deemed as a prebiotically plausible amino acid is still in doubt (9–11). Crucially, no relationship between RNA sequence and peptide sequence (or coding) has been demonstrated with these systems. Two examples of RNA-templated peptide synthesis, in a nicked duplex or across a duplex terminus, were published recently (12,13). A condensation buffer containing an excess of a conventional chemical activating agent, rather than a prebiotically plausible one, was required in either case, as the coupling reactions do not appreciably occur without them. Furthermore, an adequate hypothesis for how either of these mechanisms could have transitioned to the extant ribosomal mechanism over time is yet to be proposed. As such, a question remains over their relevance to the origin and early evolution of life.

**Figure 1.**
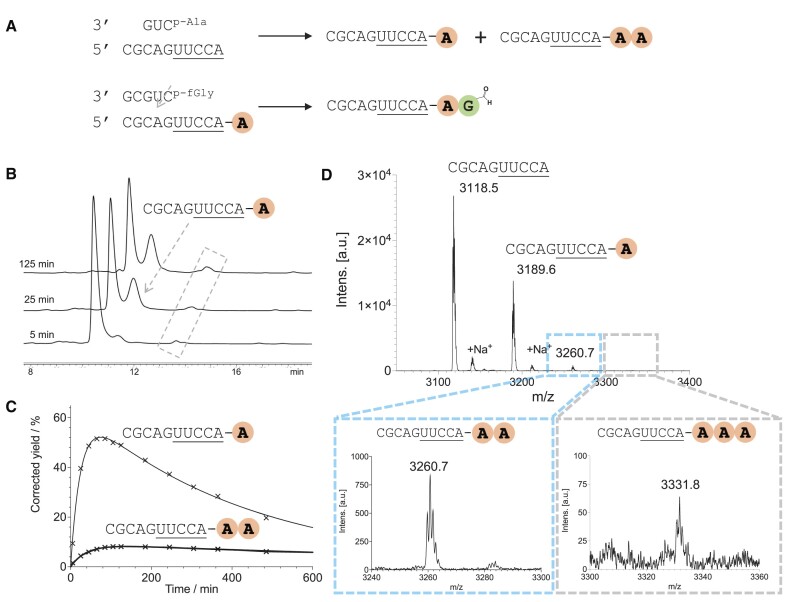
Peptide synthesis via alanyl mixed anhydride transfer. (**A**) Schematic representation of aminoacyl transfer; (**B**) HPLC trace of alanyl mixed anhydride transfer over time. The broken box shows a new peak other than the 10-mer acceptor strand and its 2′/3′-Ala-esters, (indicated by the dashed arrow); (**C**) time courses of the 2′/3′-Ala-esters (thin line) and the 2′/3′-dipeptidyl-esters (putatively) corresponding to the new peaks over time (corrected for the mixed anhydride yield); (**D**) MALDI-TOF results for the aminoacyl-transfer reaction, 2′/3′-Ala-Ala-esters, calculated 3260.6, found 3260.7; 2′/3′-trialanyl-esters, calculated 3331.7, found 3331.8. A and G in bold and coloured circles indicate amino acids alanine and glycine. Conditions: acceptor strand 100 μM, Ala-mixed anhydride donor strand (3 equivalents relative to the acceptor strand), HEPES 50 mM, NaCl 100 mM, MgCl_2_ 5 mM, pH 6.8, 16°C.

We have previously reported an aminoacyl-transfer reaction within a stem-overhang RNA structure (Figure 1A) (14). Starting from a chemically synthesised aminoacyl-phosphate mixed anhydride at the 5′-terminus of an RNA strand, in combination with a longer overhanging complementary RNA, the aminoacyl-residue is spontaneously transferred to the 3′-terminus forming a 2′/3′-aminoacyl-ester. The 5-mer UUCCA overhang was found to be the most efficient CCA-ending sequence for the transfer reaction. This mechanism has clear parallels to that of extant biology which utilises an aminoacyl adenylate mixed anhydride and aminoacyl tRNA synthetases to synthesize 2′/3′-aminoacyl-ester tRNAs. We have also reported recently how the transfer varies with RNA sequence and different amino acids and the possible consequences for the origin of the genetic code (15). Furthermore, we have demonstrated that the transfer is stereoselective for L-amino acyl residues when using D-RNA. Biology also uses these same enantiomers of bio-molecules and establishing this stereochemical relationship would be vital at the origins of translation. We were interested to see if further equivalents of mixed anhydride would allow subsequent aminoacyl transfer to the aminoacyl-ester amino group to give a 2′/3′-dipeptidyl-ester.

## Materials and methods

### General methods

Reagents and solvents were obtained from *Acros Organics*, *Santa Cruz Biotechnology*, *Sigma-Aldrich*, and were used without further purification. For solid-phase RNA synthesis, primer support 5G with a loading of 300 μmol/g was purchased from *Cytiva*. Phosphoramidites for RNA synthesis were purchased from *Link Technologies*. RNA oligomers used in this study were synthesized using an *ÄKTA* Oligopilot plus 10 (*Cytiva*) on a 20–50 μmol scale. *Mettler Toledo SevenEasy* pH Meter S20 combined with a *ThermoFisher Scientific* Orion 8103BN Ross semi-micro pH electrode was used to measure and adjust the pH to the desired value. Mass spectra were acquired on an *Agilent* 1200 LC–MS system equipped with an electrospray ionization (ESI) source and a 6130 quadrupole spectrometer (LC solvents: A, 0.2% formic acid in H_2_O; B, 0.2% formic acid in acetonitrile), or *Bruker* Ultraflex III MALDI-TOF/TOF. High-performance liquid Chromatography (HPLC) was run on Dionex Ultimate 3000 (*Thermo Scientific*) using an A*tlantis* T3, 3 μm, 4.6 × 150 mm column (unless stated otherwise). Oligonucleotide concentrations were determined by UV absorbance at 260 nm using a NanoDrop ND-1000 spectrophotometer. ^1^H- and ^13^C-nuclear magnetic resonance (NMR) spectra were acquired using a *Bruker* Ultrashield 400 Plus operating at 400.13 and 100.61 MHz, respectively. Data for ^1^H- experiments are reported as chemical shifts (relative integral, splitting, coupling constant, assignment) whilst for ^13^C- are reported as chemical shifts (assignment). Chemical shifts (*δ*) are shown in ppm. Coupling constants (*J*) are given in Hertz; s, singlet; d, doublet; q, quartet; qd, quartet of doublets; m, multiplet; br, broad signal.

### Solid-phase synthesis of RNA oligonucleotides

After automated synthesis, RNAs were first cleaved from the solid support by treating with 3 ml of a 1:1 mixture of NH_3_ aqueous solution (28% wt) and CH_3_NH_2_ ethanol solution (33% wt) at 55°C for 80 min in a tube with a sealed cap. The solid was removed by filtration and washed with 50% EtOH/H_2_O. The solutions were combined and evaporated to dryness under reduced pressure. Silyl protecting groups were removed by treating the residues with 2 ml of 1:1 mixture of triethylamine trihydrofluoride and DMSO at 65°C for 150 min in a tube with a sealed cap. After brief cooling at –32°C, Cold NaClO_4_ in acetone (50 mM, 40 ml) was added to the solution to precipitate the oligoribonucleotides. The resulting mixture was centrifuged and the RNA pellets were dissolved in water and purified by preparative HPLC then lyophilized. The purified RNA was redissolved in 2 ml of water and passed through a *Waters* Sep-Pak C18 Cartridge with 10 g sorbent. The cartridge was pre-washed with 50 ml of acetonitrile then 50 ml of water before sample loading, washed with 100 ml of H_2_O and 50 ml of 20% acetonitrile aq. Eluates containing RNA were checked on a Nanodrop, combined and lyophilized. The resulting white powder was stored at -32°C for future use.

Synthesis of the protected amino acids and oligoribonucleotide mixed anhydrides, transfer reaction, yield calculation and kinetic regression analysis were adapted from previous methods (14). Hydrolysis was executed for at least one half-life. MALDI-TOF characterization was conducted after desalting the reaction mixture with a Sep-Pak C18 column. See Table S1 for oligonucleotides used in this study.

### Synthesis of alanine 5-(*4H*)-oxazolone

*N*-Formyl-alanine (23.4 mg, 0.20 mmol, 1 eq.) was dissolved in dichloromethane (3 ml) and cooled to 0°C. 1-(3-Dimethylaminopropyl)-3-ethylcarbodiimide hydrochloride (EDC, 42.0 mg, 0.22 mmol, 1.1 eq.) was added and the reaction was stirred overnight. The solution was dried *in vacuo* and used directly with no further purification.

^1^H NMR (400 MHz, CDCl_3_) *δ* = 7.50 (1H, d, *J*= 2.5 Hz, H2), 4.09 (1H, qd, *J*= 7.6, 2.5 Hz, α), 1.40 (3H, d, *J*= 7.6Hz, β).

^13^C NMR (100 MHz, CDCl_3_) *δ* = 178.2 (C1), 152.9 (C2), 58.3 (α), 16.3 (β).

### Synthesis of *N*-Boc-alanyl alanine

Alanyl alanine (240 mg, 1.5 mmol) was dissolved in aqueous sodium hydroxide solution (1 M, 2.9 ml) and tetrahydrofuran (2.0 ml) and cooled on ice. Ditertbutyl dicarbonate (393 mg, 1.8 mmol) was dissolved in tetrahydrofuran (THF, 1.6 ml) and added dropwise over 5 min. The reaction was stirred on ice for 30 min before being stirred overnight at room temperature. The solution was cooled to 0°C and acidified to pH = 4 with aqueous HCl (2 M). The aqueous layer was extracted with diethyl ether (20 ml × 3). The combined organic fractions were dried with magnesium sulphate and concentrated to dryness *in vacuo*. The solid was recrystallised from diethyl ether affording 100 mg (26% yield) of colourless needle-like crystals.

^1^H NMR (400 MHz, CDCl_3_) *δ* = 6.97 (1H, d, *J*= 7.2 Hz, NH), 5.27 (1H, s, NH), 4.56 (1H, q, *J*= 7.2 Hz, α1), 4.24 (1H, br, α2), 1.45 (12H, m, β1 + Boc), 1.36 (3H, d, *J*= 7.1 Hz, β2).

^13^C NMR (100 MHz, CDCl_3_) *δ* = 210.9 (CO-Boc), 175.6 (CONH), 173.1 (CO_2_H), 80.3 (Boc (C(CH_3_))), 50.1 (α2), 48.3 (α1), 28.4 (Boc (CH_3_)) 18.3 (β2), 18.1 (β1).

### Synthesis of *N*-protected aminoacyl-/peptidyl- adenosine

Adenosine (1.3 mg, 4.9 μmol, 1.0 eq.) was dissolved in a mixture of H_2_O (375 μl), D_2_O (50 μl) and DMSO (25 μl) with the aid of a heat gun. Carbonyl diimidazole (CDI, 4.4 mg, 27.4 μmol, 5.6 eq.) and the desired *N*-protected amino acid or Boc-Ala-Ala (27.4 μmol, 5.6 eq.) were dissolved in acetonitrile (250 μl) and vortexed for 3 min at room temperature. An aliquot of the amino acid solution (50 μl) was added to the adenosine solution. The mixture was shaken (300 rpm) for 10 min at room temperature. If the amino acid was Boc protected, the solution was acidified to pH 4 with aqueous HCl (1 M), then concentrated *in vacuo*. Trifluoroacetic acid (200 μl) was added and the mixture was left to stand at room temperature for 10 min before being concentrated *in vacuo* and redissolved in a mixture of H_2_O (450 μl), D_2_O (50 μl). If the amino acid was formyl protected, solutions were taken on directly. All samples were analysed both by HPLC with 260 nm UV detection. Solvent A: 25 mM TEAA (triethylamine buffered to pH 4.5 with acetic acid); solvent B: acetonitrile; flow rate 1 ml/min; column compartment 25°C; gradient 0 min, 0%; 18 min, 25%; 19 min, 85%; 22 min 85%; 23 min, 0%; 26 min, 0%. For formyl-glycylalanyl ester adenosine, a dichloromethane solution (5 ml) of *N*,*N*′-dicyclohexyl-carbodiimide (DCC, 107 mg, 0.52 mmol, 2 eq.) and formic acid (20 μl, 0.52 mmol, 2 eq.) were stirred at room temperature for 5 min before glycyl alanine (*Santa Cruz*, sc-479092, 38 mg, 0.26 mmol, 1 eq.) was added. The mixture was further stirred at room temperature for 8 h. The precipitate was filtered, and the dichloromethane solvent was removed. The residue was redissolved in acetonitrile (500 μl) and stirred with CDI (16.9 mg, 0.10 mmol, 0.4 eq.) for 3 min before an aqueous suspension (2 ml) of adenosine (13.9 mg, 0.05 mmol, 0.2 eq.) was added. The mixture was further stirred for 10 min while monitoring the pH to 7.0, before being quenched with 10% formic acid. The mixture was purified using HPLC. Solvents as above, flow rate 5 ml/min. See Supplementary Figures S23–S30 for ^1^H NMR spectrum for the above adenylates.

### RNase A digestion

When necessary, samples of a desired retention time were collected from analytical HPLC and then lyophilized. Sodium acetate solution (pH 4.0, 0.5 M, 1 μl) was added to either an aliquot of crude reaction (9 μl) or redissolved lyophilizate (9 μl, H_2_O). RNase A (1 μl, 10 mg/ml, *Thermo Fischer*, EN0531) was added and the solution was shaken for 30 min (20°C, 300 rpm). Methanol (11 μl) was added and the mixture was centrifuged for 30 min (4°C, 21 000 × g). The supernatant was recovered and analysed by HPLC with *Atlantis* T3, 5 μm, 4.6 × 250 mm column. Solvents and gradients as above.

## Results

In the HPLC trace of transfer reaction of alanyl (Ala) residues (3 eq. 5′-L-Ala-pCUG mixed anhydride as donor strand mixed with 1 eq. 5′-CGCAGUUCCA as acceptor stand, Figure 1A, see supplementary data for schematic representations) we observed a new peak with a longer retention time than that of the 2′/3′-Ala-ester acceptors (Figure 1B). The peak reached its maximum yield of 8% after 140 min, compared to 70 min for the known 2′/3′-Ala-esters (Figure 1C, Supplementary Table S2, Supplementary Figure S1). This new peak decreased in intensity slowly with a half-life of 14 h at 16°C, more than double that of the 2′/3′-Ala-esters. After isolation of species responsible for the new peak, MALDI-TOF characterization was consistent with a 10-mer strand plus two Ala residues (Figure 1D). Although it is possible in theory to produce a bis-2′,3′-Ala,Ala-diester (with Ala residues both at terminal or internal 2′ and terminal 3′ positions), we would expect such species to have comparable lability to the 2′/3′-Ala-esters. However, we expected the 2′/3′-dialanyl-esters (Ala-Ala) to be more stable than the monoalanyl esters and therefore tentatively assigned the new peak to result from the formation of the 2′/3′-Ala-Ala-ester acceptor strands.

We had previously used RNase A digestion to analyse *N*-acetyl-aminoacylation of RNA (14). RNase A cleaves the terminal adenosine nucleoside of the acceptor stem whilst maintaining any aminoacyl ester bonds. By comparison against synthetic standards, the 2′/3′-(*N*-acetyl)-Ala-ester adenosines could be identified in the digestion products of *N*-acetyl-aminoacylated-RNA when analysed by HPLC, and thus the presence of terminal 2′/3′-*N*-acetyl-aminoacyl-esters in the undigested RNA could be deduced. To provide further verification that the new peak we had observed in the current work indicated the formation of 2′/3′-Ala-Ala-esters, we HPLC purified the species responsible for the peak, then lyophilised and digested it with RNase. The chromatogram of the digested material contained peaks consistent with both 2′- and 3′-Ala-Ala-ester adenosines (Supplementary Figure S2, Supplementary Figure S3) when compared to synthetic standards. The region of the chromatogram corresponding to the bis-2′,3′-Ala,Ala-diester adenosine standard was quite congested in the digested sample, but very low in intensity. We conclude the new peak to result from the formation of 2′/3′-Ala-Ala-esters. Furthermore, we were able to identify a mass peak corresponding to the 10-mer strand plus three alanine residues by MALDI (Figure 1D), which is logically even more likely to include at least one peptide bond.

**Figure 2.**
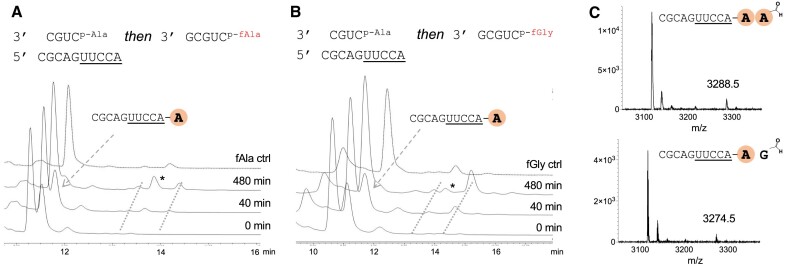
Peptide synthesis via transfer of an alanyl residue followed by *N*-formyl-aminoacyl transfer. HPLC traces of (**A**) fAla and (**B**) fGly transfer to 2′/3′-Ala-ester acceptors. The dotted lines show two new peaks corresponding to 2′/3′-*N*-formyl-dipeptidyl-ester acceptors over time. fAla and fGly ctrl indicate the reaction of fAla-/fGly-mixed anhydride donor transferred directly to the 2′/3′-diol of a 10-mer acceptor to give 2′/3′-fAla/fGly-esters. * indicates 2′/3′-fAla/fGly-esters. (**C**) The respective MALDI spectra and data. A and G in bold and coloured circles indicate amino acids alanine and glycine. Conditions: acceptor strand 100 μM, Ala-mixed anhydride donor strand (1 eq.) and *N*-formyl-aminoacyl mixed anhydride donor strand (2 eq.) to the acceptor strand, HEPES 50 mM, NaCl 100 mM, MgCl_2_ 5 mM, pH 6.8, 10°C. fAla/fGly ctrl was performed at pH 8.0. Stacked HPLC traces are staggered by 15 s each for clarity.

In prokaryotes, peptide synthesis starts with *N*-formyl-methionine (fMet) encoded by the start codon AUG. The formyl group allows the corresponding aminoacyl-tRNA to be accommodated at the P site of the ribosome. *N*-formyl-amino acids have been demonstrated to be prebiotically plausible species on early Earth (16). Therefore, we studied *N*-formyl-alanyl (fAla) and *N*-formyl-glycyl transfer (fGly) (Figure 2A, B, Supplementary Table S3, Supplementary Figure S4, Supplementary Figure S5). First, we demonstrated that both fAla and fGly themselves directly transferred to the 3′-termini of a 10-mer acceptor bearing no other aminoacyl residues (maximum observed yields 16% and 21% at pH 8.0, Figure 2A, B ctrl lines). Next, we tested whether a sequential addition could form peptidyl-RNA. Starting with the same sequence 10-mer acceptor strand, an Ala residue was first transferred to the 10-mer 3′-terminus using an alanyl mixed anhydride-tetramer donor (5′-L-Ala-pCUGC). When the yields of 2′/3′-Ala-esters reached their peaks, 2 eq. fAla- or fGly-mixed anhydride-pentamer donor (5′-fAla-/fGly-pCUGCG) was added to the system.

After the addition of *N*-formyl-aminoacyl mixed anhydride, the peak corresponding to the 2′/3′-Ala-esters was observed to decrease much faster (half-life = 1.9 h for fGly and 3.3 h for fAla) than without the *N*-formyl-aminoacyl mixed anhydride (half-life = 6.6 h). New peaks with similar kinetic profiles to each other were detected (Supplementary Figure S4, Supplementary Figure S5) as well as peaks identifiable as 2′/3′-*N*-formyl-aminoacyl-esters by comparison with a control reaction (Figure 2A, B ctrl lines). After isolation, the species responsible for the new peaks produced MALDI peaks consistent with RNA plus both Ala- and fGly/fAla- residues (Figure 2C). Despite our previous assumption that no bis-2′,3′-products were formed, we entertained the possibility these new peaks may correspond to the 2′,3′-Ala-,fAla-/Ala-,fGly-diesters. We would expect that both 2′,3′-Ala-,fAla- and 2′,3′-Ala-,fGly-diesters would first hydrolyse to 2′/3′-fAla- or 2′/3′-fGly-esters. However, the corresponding peaks for these species did not increase as the new peaks degraded, supporting the formation of 2′/3′-dipeptidyl esters instead.

As there was always a substantial proportion of unreacted 10-mer acceptor strand after the first step, we also observed the direct formation of 2′/3′-fAla-/fGly-esters from said acceptor. Due to the increased nucleophilicity of a 2′,3′-diol compared to a single hydroxyl, we would expect more 2′/3′-fAla-/fGly-ester to form than any bis-species. However, in the case of fAla transfer (Figure 2A, Supplementary Table S3), we found the rate constants of formation of 2′/3′-fAla-ester and the new species to be nearly identical (0.0066 and 0.0067 min^–1^ respectively). In the case of fGly transfer (Figure 2B, Supplementary Table S3), the integration of the new peaks was 2-fold higher than the integration of the 2′/3′-fGly-ester peak. All these observations suggest that the new peaks did not correspond to bis-species, but instead indicated the formation of 2′/3′-formyl-dipeptidyl-esters.

We further studied the transfer by RNase A digestion of the products of fGly transferred onto preformed 2′/3′-Ala-esters. By comparison of the HPLC chromatograms of the digestion products with synthetic standards, we identified peaks corresponding to the 2′/3′-fGly-Ala-esters of adenosine, and none of the bis-2′,3′-Ala-,fGly-diesters, demonstrating that the transfer reaction does not produce bis-2′,3′-diesters. (Supplementary Figure S3, Supplementary Figure S6)

In the case of the fAla transfer onto preformed 2′/3′-Ala-esters, the question remained, why were there two new peaks with significant differences in retention time? When a fAla-residue was transferred from a pentamer donor to the 3′-terminus of the 10-mer acceptor as a control experiment, two overlapping peaks were observed (Figure 2A). This could be consistent with racemization of fAla during the chemical synthesis of the mixed anhydride followed by transfer of both D- and L-fAla-residues. Previous experiments had demonstrated that D-alanyl mixed anhydrides would only transfer inefficiently whilst D-*N*-acetyl-alanyl (D-AcAla) mixed anhydrides transferred equally well as L- across a nicked loop (14,15). Alternatively, the two new peaks could be due to a mixture of 2′/3′-aminoacyl-esters resolving differently on the column. In the case of fGly transfer to a 2′,3′-diol, only one peak was identified, which could suggest either the transfer was regioselective for one alcohol or that both 2′/3′-aminoacyl-esters have equal retention times.

The yield of peptide bond formation differed between these formylaminoacyl residues (2′/3′-fAla-Ala-esters 23%, 2′/3′-fGly-Ala-esters 57%). Unsurprisingly, due to the difference in charge state, 2′/3′-fAla-Ala-esters were less labile to cleavage (*t*$\frac{1}{2}$ = 4.1 days at 10°C, pH 6.8) than 2′/3′-Ala-Ala-esters (*t*$\frac{1}{2}$ = 1 day at 10°C, pH 6.8) which (as well as direct hydrolysis by hydroxide) can additionally cleave to form diketopiperazines (see ESI for schematic representations). The half-life of the 2′/3′-fGly-Ala-esters under the same conditions was *t*$\frac{1}{2}$ = 3.4 days, a bit shorter than the 2′/3′-fAla-Ala-esters.

**Figure 3.**
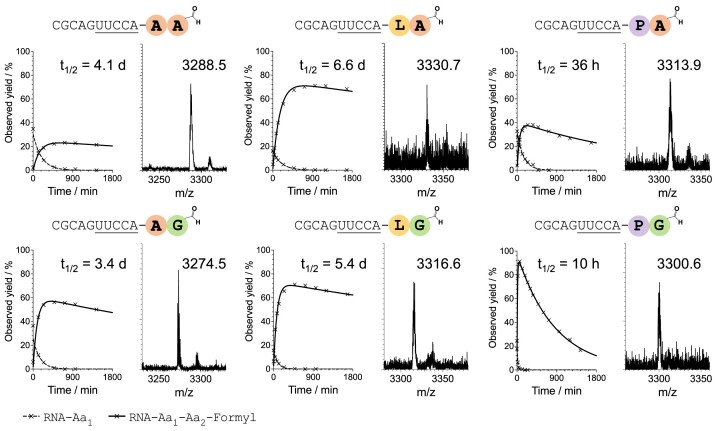
Time courses and MALDI characterization of 2′/3′-*N*-formyl-dipeptidyl RNA. Timepoint 0 was set at the peak yield of 2′/3′-aminoacyl-esters at which point the formylaminoacyl-mixed anhydride donor was added. The solid line in the time course represents the percentage yield of 2′/3′-fGly-/fAla-dipeptidyl-esters relative to the 2′/3′-aminoacyl-esters. The broken line represents the consumption due to hydrolysis and formylaminoacylation of the 2′/3′-aminoacyl-esters. A, G, L, P in bold and coloured circles indicate amino acids alanine, glycine, leucine, and proline. Conditions: acceptor strand 100 μM, aminoacyl-mixed anhydride tetramer donor strand (1 eq.) and *N*-formyl-aminoacyl mixed anhydride pentamer donor strand (2 eq.) to the acceptor strand, HEPES 50 mM, NaCl 100 mM, MgCl_2_ 5 mM, pH 6.8, 10°C.

Next, we broadened our aminoacyl mixed anhydride scope in the first step to include leucyl- and prolyl-residues (Leu and Pro respectively). For the second transfer, fAla- or fGly-mixed anhydride pentamer donor strand was used as with previous experiments (Figure 3, Supplementary Table S3, Supplementary Figure S7-S10). Transfers of fAla onto Leu- or Pro-acceptors produced four new HPLC peaks with identical kinetics, whilst transfers of fGly onto Leu or Pro produced two new ones. This is consistent with both the racemization of fAla and a resolution of the mixture of 2′/3′-esters in the products. We did not further differentiate these peaks. HPLC and MALDI analysis confirmed that all four 2′/3′-formyl-dipeptidyl-esters were correctly synthesized, with distinct yields and synthesis/hydrolysis kinetics. In the first step, Ala transferred better than Leu and Pro as reported previously (15). In the second step, fGly generally transferred better or as well as fAla, depending on the identity of the first amino acid transferred.

Both the constituent amino acids in the formyl-dipeptidyl-ester affected the ester lability, despite their inability to form diketopiperazines. It has previously been shown that a Leu-tRNA ester was more stable than an Ala-tRNA ester or a Pro-tRNA ester (17). We observed the same trend. Furthermore, the 2′/3′-formyl-dipeptidyl-esters containing fAla are always found to be more stable than those containing fGly.

The duplex-overhang 2′/3′-formyl-dipeptidyl-esters are equivalent to the acceptor stem of a peptidyl-tRNA. In extant translation, releasing the mature peptides is assisted by the Gly-Gly-Gln motif in the Class I release factors (18). But hydrolysis of 2′/3′-peptidyl-esters in a prebiotic context has not been documented to the best of our knowledge. In this work, with the help of aminoacyl transfer chemistry, we have built a small but diverse series of 2′/3′-(*N*-formyl)-dipeptidyl-esters and characterized their hydrolytic stability (Table S3). The longest half-life among the six 2′/3′-formyl-dipeptidyl-esters is 2′/3′-fAla-Leu-ester (*t*$\frac{1}{2}$ = 6.6 d at 10°C, pH 6.8). This suggests that under these conditions peptidyl RNA conjugates can form, and become enriched without auxiliary macromolecules.

In all three examples where fGly-mixed anhydride was used in the second transfer, we identified two peaks with longer retention time and greater stability toward hydrolysis (peaks marked with * in Supplementary Figure S5, Supplementary Figure S8, Supplementary Figure S10). These peaks may correspond to 2′/3′-tripeptidyl-esters terminating in fGly. This would require fGly-mixed anhydride to react with the 2′/3′-dipeptidyl-esters we identified at the beginning of this communication (Figure 1B, Supplementary Figure S1). Due to the lack of material, we were unable to characterize these peaks further.

**Figure 4.**
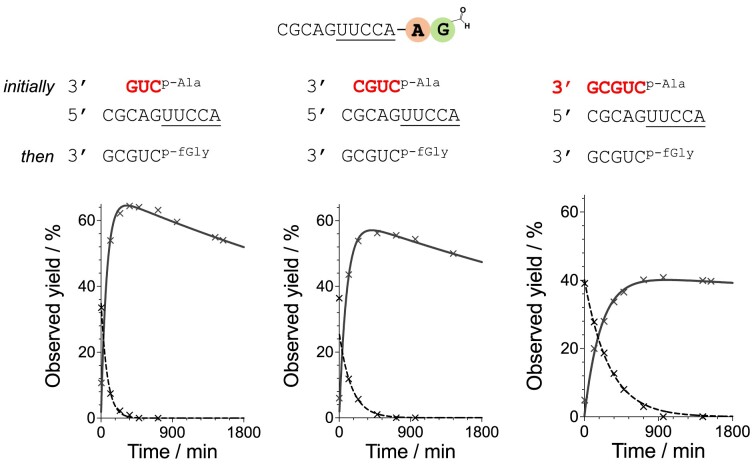
Peptidyl-RNA synthesis is achieved by donor strand replacement. Time courses for the formation of 2′/3′-fGly-Ala-esters using different length Ala-mixed anhydride donor strands. A and G in bold and coloured circles indicate amino acids alanine and glycine. Conditions: acceptor strand 100 μM, Ala-mixed anhydride initial donor strand (1 eq.), *N*-formyl-glycyl-mixed anhydride donor strand (2 eq.) added at the peak formation of 2′/3′-Ala-esters, HEPES 50 mM, NaCl 100 mM, MgCl_2_ 5 mM, pH 6.8, 10°C.

We investigated the mechanism of the multi-step *N*-formyl-aminoacyl transfer. We prepared the alanyl mixed anhydride donor in three different lengths, trimer (5′-L-Ala-pCUG), tetramer (5′-L-Ala-pCUGC) and pentamer (5′-L-Ala-pCUGCG). As the first transfer reached its maximum, two equivalents of a fGly-mixed anhydride pentamer (5′-fGly-pCUGCG) were added. With the increasing length of the original alanyl-mixed anhydride donor, it becomes harder for the fGly-mixed anhydride pentamer donor to replace it, thus reducing the yield and rate of dipeptidyl ester formation. The 5′-L-Ala-pCUG followed by 5′-fGly-pCUGCG transfer gave the highest dipeptide yield (65%) in the shortest time (325 min) (Figure 4, Supplementary Table S4). The 5′-L-Ala-pCUGC followed by 5′-fGly-pCUGCG transfer yield was 57% (at 410 min), whilst the 5′-L-Ala-pCUGCG followed by 5′-fGly-pCUGCG transfer was the slowest, reaching to maximum yield of 40% after 1000 min. These data indicate the necessity of strand exchange before *N*-formyl-dipeptidyl ester formation.

When *N*-acyl amino acids are activated at their C-termini they form oxazolones (see Supplementary Figure S11-S15 and Supplementary Figure S22 for relevant structures) which are reactive electrophiles (19). It is possible the formyl-aminoacyl-mixed anhydrides could undergo reversible cleavage from 5-(*4H*)-oxazolones, which then subsequently react intermolecularly with the nucleophilic amine of the 2′/3′-aminoacyl-ester. To test this hypothesis we incubated the 10-mer acceptor strand and a pentamer (pCUGCG) containing a 5′-phosphate then added the water-soluble carbodiimide EDC and fGly/fAla to provide *in situ* formation of oxazolone (Supplementary Figures S11 and S12). This failed to produce any acyl transfer products, mixed anhydride or other RNA esters. Repeating the reaction instead using preformed Ala 5-(*4H*)-oxazolone up to 2000 eq. also failed to produce any peaks where we would expect transfer products nor mixed anhydride, in contrast to previous studies with nucleosides (19) (Supplementary Figures S13 and S14). Similar addition of Ala 5-(*4H*)-oxazolone to preformed 2′/3′-Ala-esters did not produce new peaks with the correct retention time or stability (Supplementary Figure S15). As no transfer to the 10-mer strand is observed at such high concentrations of oxazolone and given the relative stability of the formyl-aminoacyl-mixed anhydrides under these conditions, we think it unlikely that 5-(*4H*)-oxazolone is implicated in the formation of *N*-formyl-dipeptidyl-2′/3′-esters or *N*-formyl-aminoacyl-2′/3′-esters presented above.

As diastereoselectivity of transfer had only been studied for the transfer of AcAla residues, we studied the transfer of the more prebiotically plausible fAla residues used in this work. A 5-mer mixed anhydride synthesised from L-fAla as a donor strand was mixed with a 10-mer acceptor strand (1 eq. 5′-fAla-pAGCGA mixed anhydride with 1 eq. 5′-UCGCUUUCCA forming a nicked loop) or with a 10-mer template strand and 8-mer acceptor strand (1 eq. 5′-fAla-pAGCGA mixed anhydride with 1 eq. 5′-UCGCUUUCCA template and 1 eq. 5′-UAAUGGAA acceptor making a nicked duplex). fAla transfer to their acceptors was identified across a nicked loop (Supplementary Figure S16, 16%) or nicked duplex (Supplementary Figure S17, 61%) by the presence of new peaks with increased retention time consistent with our previous experiments. The crude mixtures were digested by RNase A as before and analysed by HPLC (Supplementary Figures S18 and S19). Comparison of these chromatograms with those of synthetic standards confirmed the presence of nearly equal amounts of both L- and D-2′/3′-fAla-ester adenosines. These results were consistent with racemization of the formylalanyl residue during synthesis of the fAla mixed anhydride 5-mer, followed by the transfer of both L- and D-fAla residues. Repeating these experiments, but starting with D-fAla, afforded similar transfer yields (13% nicked loop and 50% nicked duplex, Supplementary Figures S16 and S17) and resulted in nearly identical chromatograms (Supplementary Figures S18 and S19).

At this point, it seemed odd that only one out of the four possible combinations of *N*-acetyl-, and *N*-formyl-aminoacyl transfers across either a nicked-loop or nicked-duplex was stereoselective. We synthesised 5-mer mixed anhydrides using either L- or D-AcAla as a donor strand, then added them to a nicked duplex scenario containing the same 10-mer template strand and 8-mer acceptor strand as our earlier experiments (1 eq. 5′-AcAla-pAGCGA mixed anhydride with 1 eq. 5′-UCGCUUUCCA template and 1 eq. 5′-UAAUGGAA acceptor). We identified the transfer of AcAla residues across a nicked duplex by formation of a new peak as before (starting from Ac-L-Ala 25%, starting from Ac-D-Ala 22%; Supplementary Figure S20). The species responsible for this peak were collected, lyophilised and then digested with RNase. Subsequent HPLC analysis and comparison to synthesised standards demonstrated the presence of equal amounts of L- and D- 2′/3′-AcAla-ester adenosines (Supplementary Figure S21).

## Discussion

Oxazolones are known to racemize (20). The synthetic chemistry used to make formylaminoacyl mixed anhydrides in this work would likely produce 5-(*4H*)-oxazolones as reactive intermediates, so we would expect both diastereoisomers of the formyl-aminoacyl mixed anhydrides to be formed. Stereoselectivity of transfer of *N*-acyl-aminoacyl residues would have been important to nascent translation to ensure diasteromerically pure proteins. We and others have previously demonstrated that the transfer of aminoacyl residues from a mixed anhydride in a nicked loop (14) or nicked duplex (21) configuration is stereoselective with some exceptions for specific sequences (15). However, nicked loop transfer of AcAla residues is not stereoselective (14). This last observation is interesting in comparison to a previous report that AcAla residues do in fact transfer stereoselectively in a nicked duplex (22).

In contrast to the previous report, we conclude that *N*-acyl-aminoacyl residues do not transfer stereoselectively in either nicked-loop or nicked-duplex configurations. Due to the lack of experimental detail reported by Tamura *et al.* (22), it is difficult to see where our conflicting results originate. It is possible that the different sequences used demonstrate different levels of selectivity, something we have seen in some exceptional cases with the transfer of aminoacyl residues in a nicked loop (15). Alternatively, simple experimental errors in the previous work such as differing loading onto gels (no loading control was shown) or significantly hydrolysed mixed anhydride starting material synthesised from D-AcAla, are potential causes.

Our results here demonstrate the possibility of assembling prebiotic peptidyl-RNA via strand replacement and transfer from *N*-formyl-aminoacyl mixed anhydrides. We note dipeptides have been shown to have sequence specific activities relevant to the origins of life, such as vesicle growth (23). In our scenarios, stable duplex and strand replacement are the prerequisites for peptidyl assembly. Here, the peptide directly forms at the CCA-ending overhang, as in extant tRNA. The hydrolytic stability of formyl-dipeptidyl-RNA varies with amino acid sequence. Our investigations into the mechanism of *N*-formyl amino acid transfer suggest that 5-(*4H*)-oxazolones are not implicated in the mechanism of transfer. The transfer of *N*-acyl amino acids onto 3′-termini in a nicked-duplex or a nicked-loop, and onto aminoacyl esters in nicked loops show no stereoselectivity. Due to the similarity in chemistry, it is conceivable that a transition from trimer-mixed anhydride donors to adenylate-mixed anhydrides which we see in modern biology is simpler than those template chemistries presented previously (12,13). Our results also highlight the difficulty of avoiding peptidyl-RNA formation in systems capable of transferring aminoacyl residues from amino acid:phosphate mixed anhydrides to the 3′-termini of RNA.

## Supplementary Material

gkae702_Supplemental_File

## Data Availability

The data underlying this article are available in the article and in its online supplementary material.
