# Supplementary material for: RNA-directed peptide synthesis across a nicked loop
Source: Nucleic Acids Res. 2024 Aug 21;52(19):11415–22. doi: 10.1093/nar/gkae702 (PMC11514466; doi:10.1093/nar/gkae702)
Supplement: gkae702_Supplemental_File [file gkae702_supplemental_file.pdf]

SUPPLEMENTARY DATA

RNA-directed Peptide Synthesis Across a Nicked Loop

Meng Su,[a,#] Sam Roberts,[a,#] and John Sutherland[a]

a. MRC Laboratory of Molecular Biology, Cambridge CB2 0QH

Schematic representations of transfer reactions in this study.

1) L-Alanine mixed anhydride transfers from a donor strand to the 3'-acceptor strand yielding 2'-L-Ala-ester or 3'-L-Ala-ester. (reported in *JACS*, **2021**, *143*, 11836)

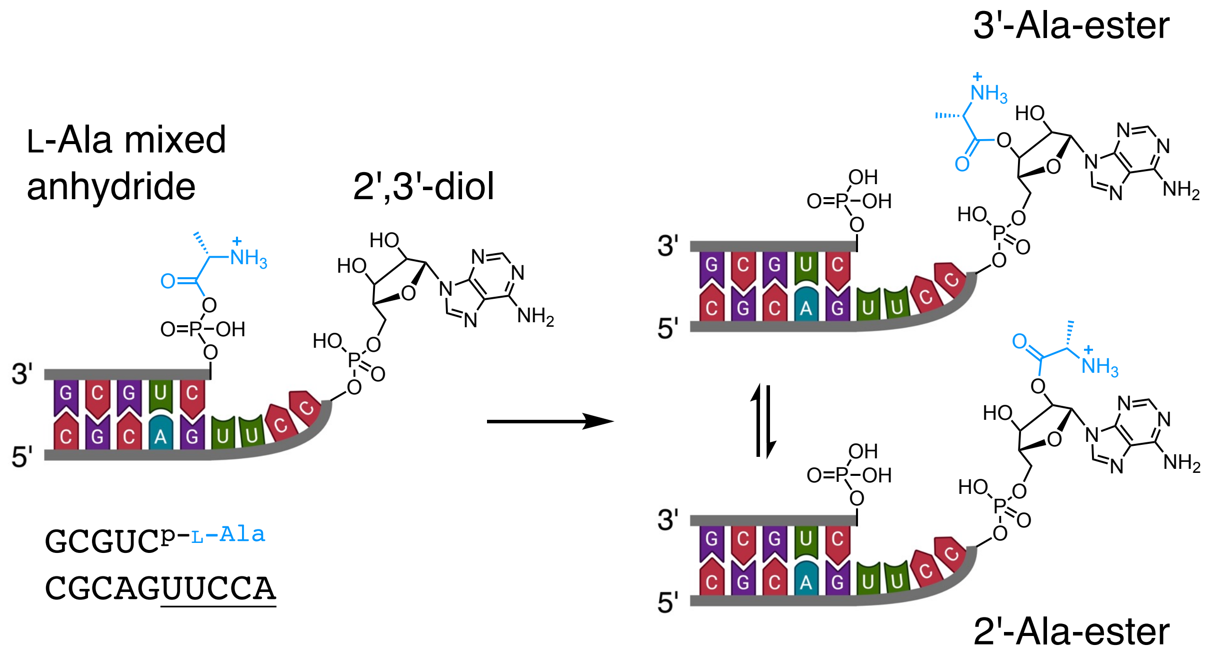

2) L-Alanine mixed anhydride transfers from a donor strand to the 2'- or 3'-L-alanyl RNA acceptor strand yielding 2'-L-Ala-L-Ala-ester or 3'-L-Ala-L-Ala-ester.

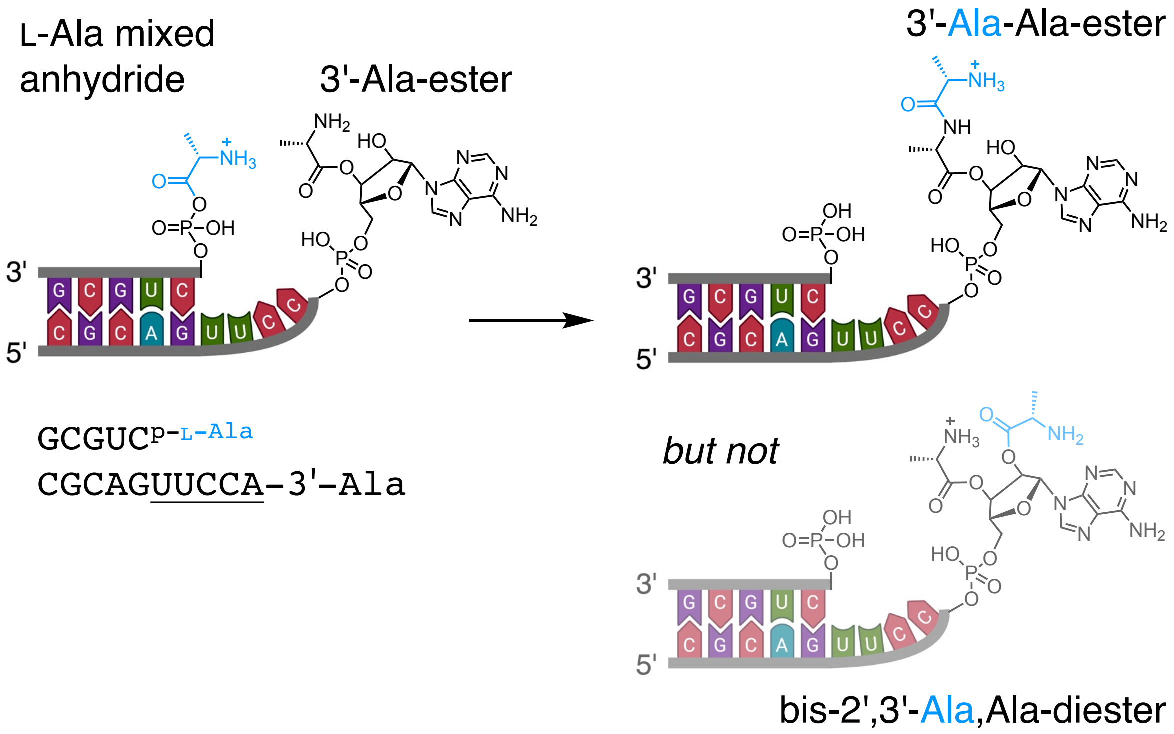

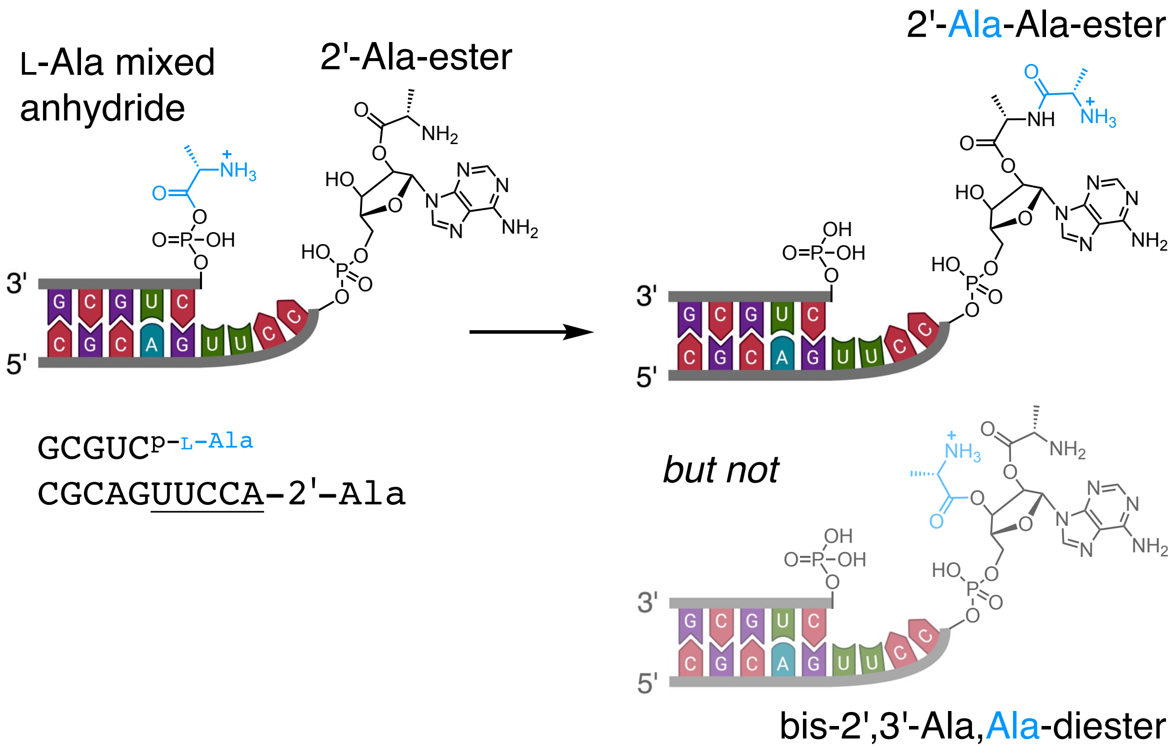

3) *N*-Formyl-alanine mixed anhydride transfers from a donor strand to the 2'- or 3'- L-alanyl RNA acceptor strand yielding 2'-fAla-L-Ala-ester or 3'-fAla-L-Ala-ester.

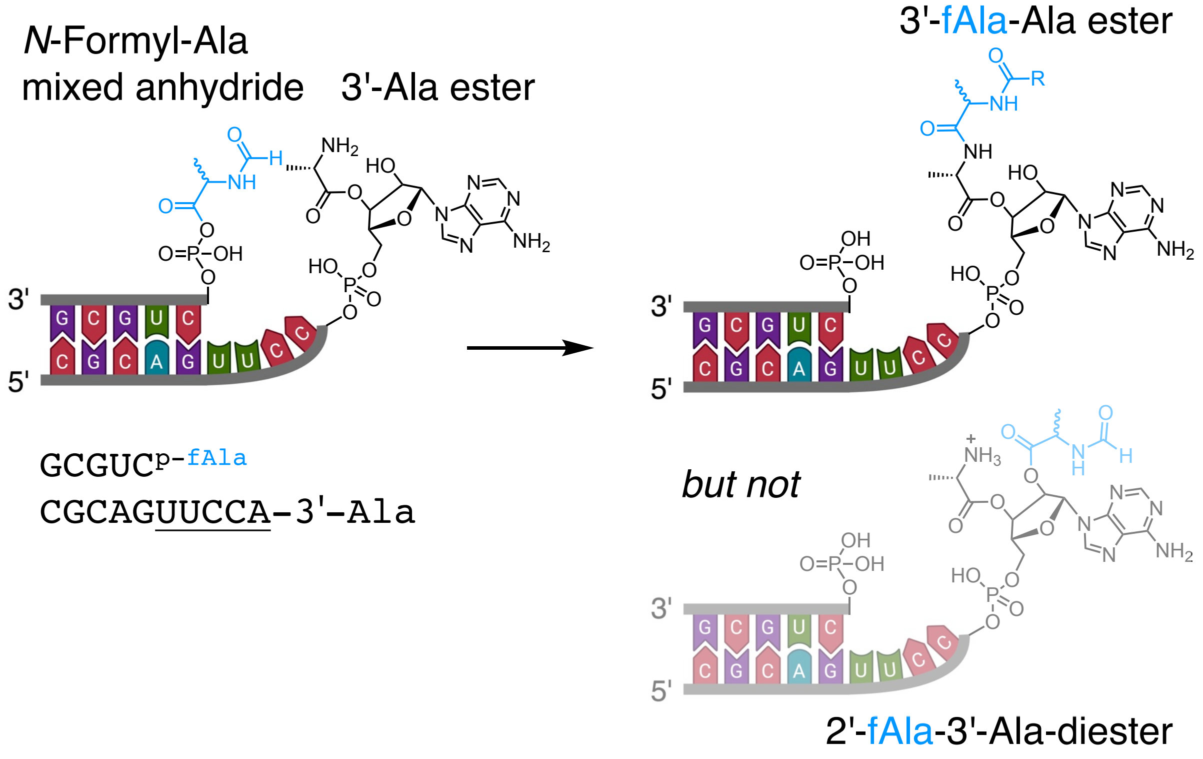

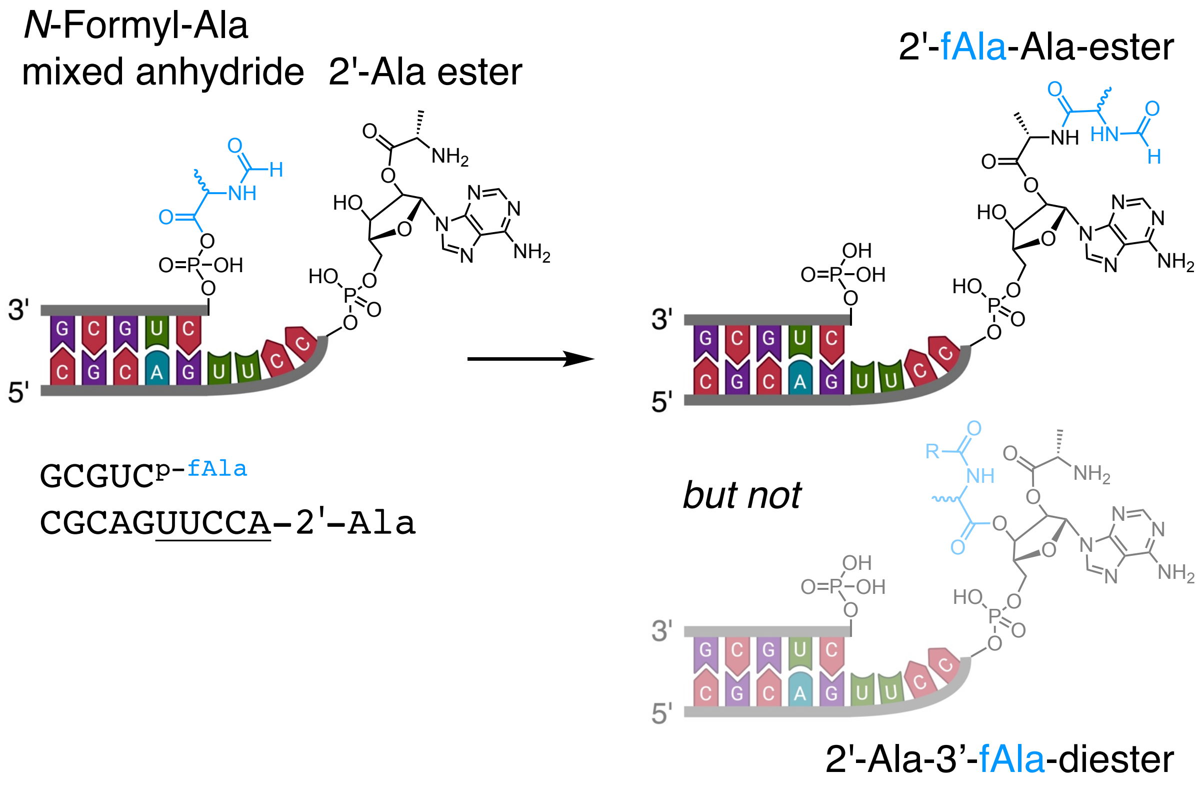

4) Internal cleavage of 2'- (or 3'-) L-Ala-L-Ala-ester, but not 2'- (or 3'-) fAla-L-Ala-ester, can occur resulting in a donor strand with 2',3'-diol, and L-Ala-L-Ala diketopiperazine (DKP). Both 2'- (or 3'-) L-Ala-L-Ala-ester and 2'- (or 3'-) fAla-L-Ala-ester can undergo hydrolysis of their ester bonds by hydroxide.

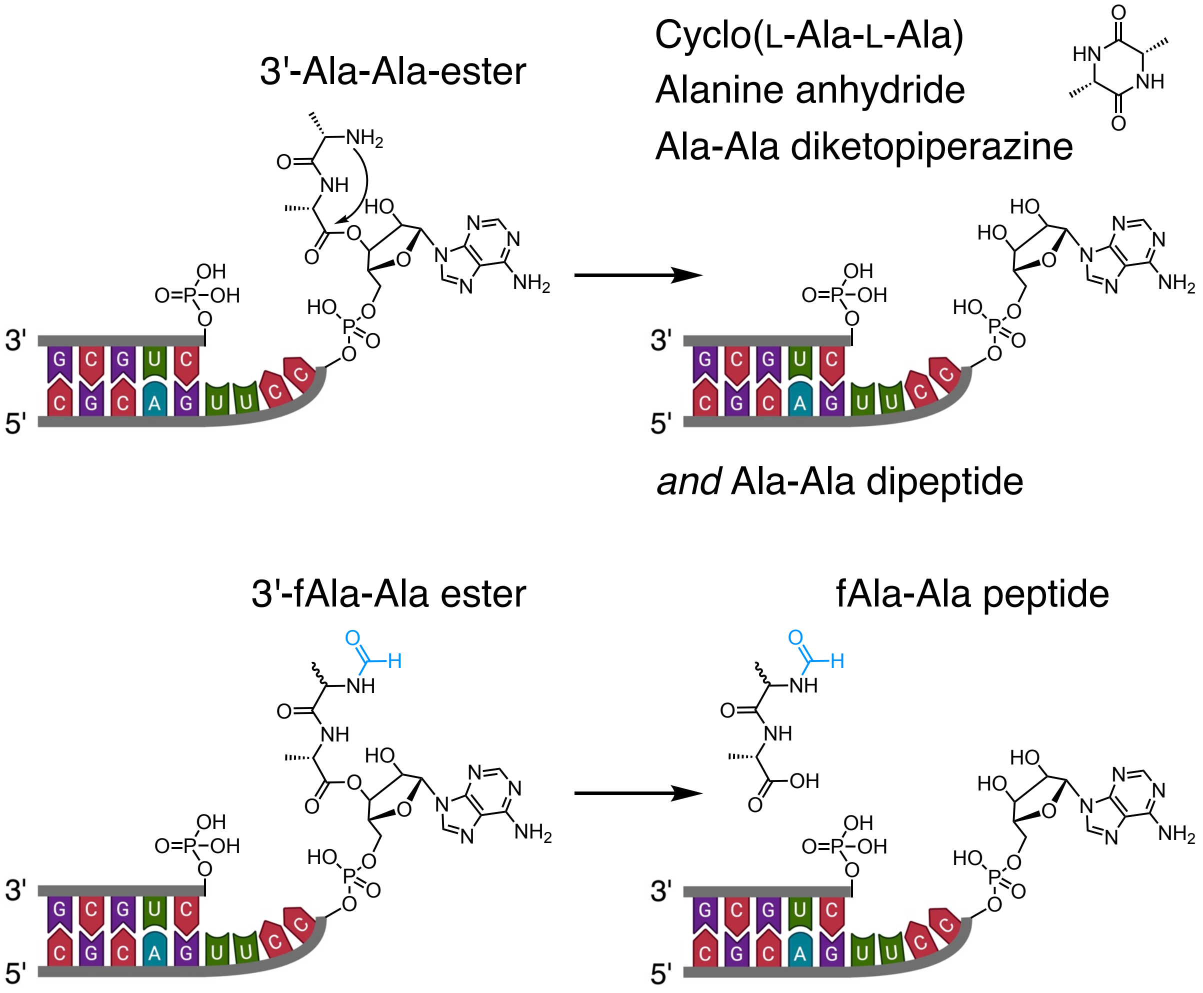

5) Summary of stereoselectivity of aminoacyl- and *N*-acyl-aminoacyl-transfer in a nicked loop, or a nicked duplex.

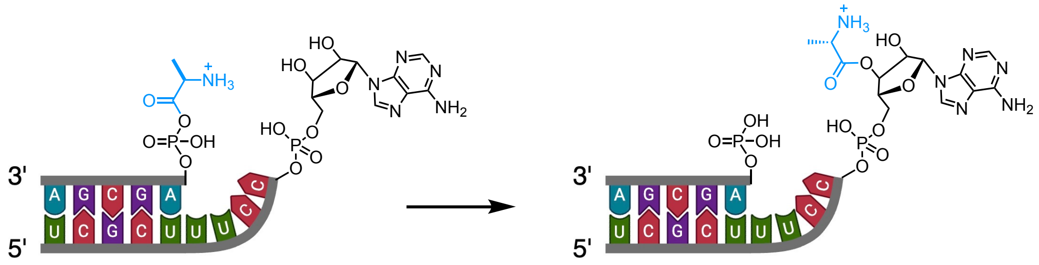

Selectively transfer L-Aa.

JACS, **2021**, *143*, 11836.

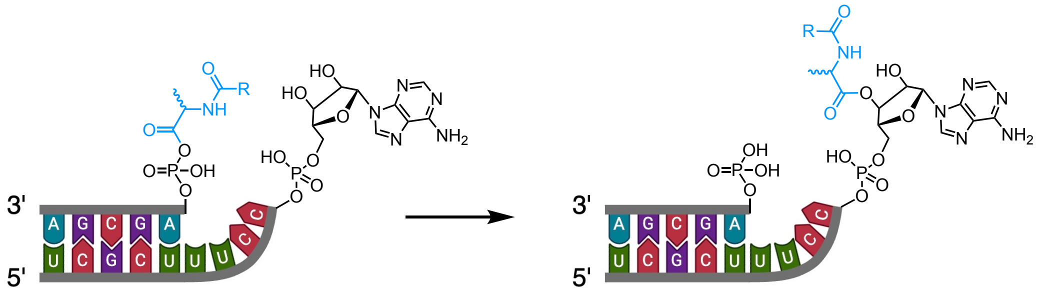

No significant stereoselectivity observed.

JACS, **2021**, *143*, 11836. (R = CH_3, UGCCA overhang)

This work (R = H, UUCCA overhang)

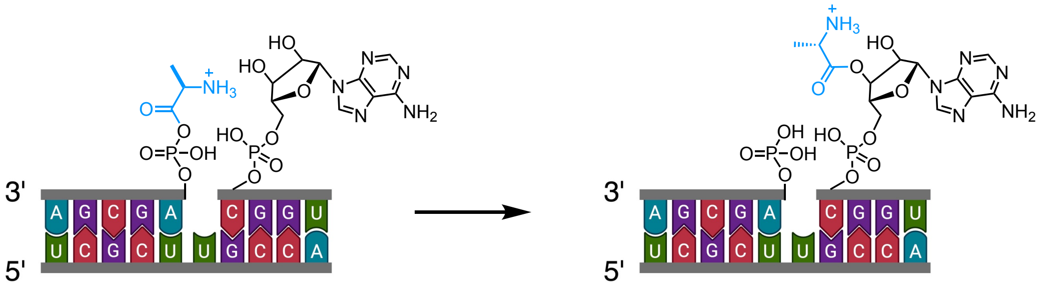

Selectively transfer L-Aa.

Science, **2004**, *305*, 1253. (dT_6dA_2 as donor strand)

JACS, **2021**, *143*, 11836.

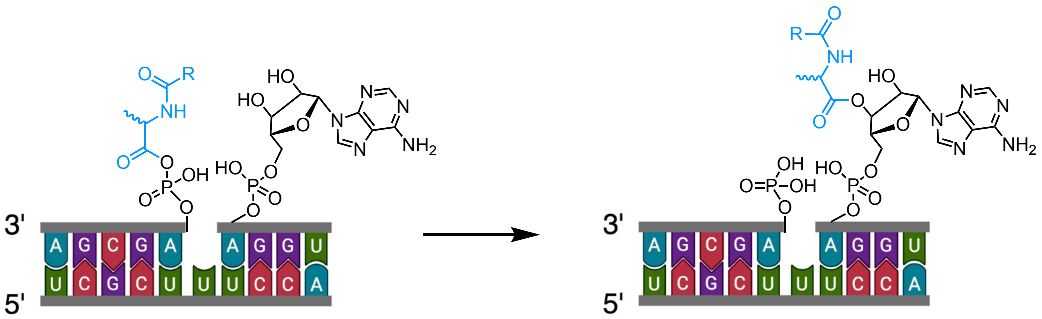

No significant stereoselectivity observed.

This work (R = H, CH_3)

6) A further example of stereoselective transfer of phenylalanyl mixed anhydride to form 5' to 2'-OH at 3'-termini.

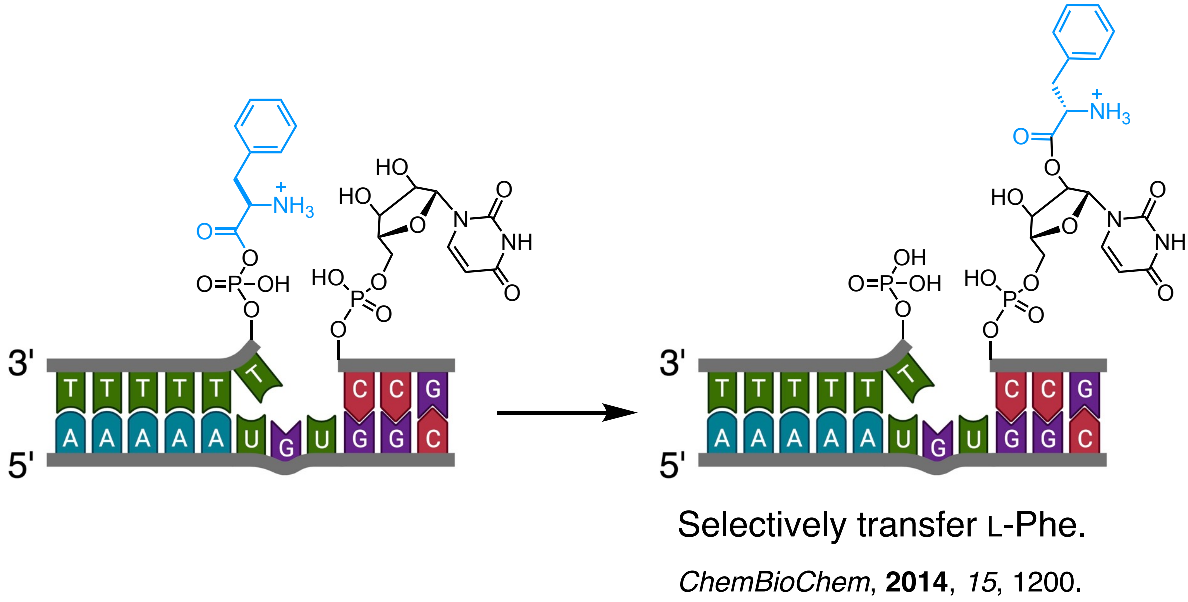

Table S1. Kinetic data of aminoacyl-transfer with 5'-L-Ala-pCUG and 5'-CGCAGUUCCA. Numbers in bold refer to 5'-CGCAGUUCCA-Ala-Ala. Conditions: 5'-CGCAGUUCCA 100 µM, HEPES 50 mM, NaCl 100 mM, $MgCl_2$ 5 mM, pH 6.8, 10°C. Ovh, overhang.

	Donor	Eq.	Acceptor		Obs. yield	Cor. yield	Diol ester half-life /h	Peak time min	k transfer /min^{-1}	k hydrolysis /min^{-1}	Notes
			Stem	Ovh							
10°C	5'-L-Ala-pCUG	1	5'-CGCAG	UUCCA	38%	46%	8.3	115	0.0272	0.00140	5'-CGCAG UUCCA-Ala
					**3%**	**4%**	**23**	**189**	**0.0199**	**0.00051**	5'**-CGCAG UUCCA-Ala-Ala**
	5'-L-Ala-pCUG	3	5'-CGCAG	UUCCA	43%	52%	7.5	75	0.0470	0.00153	5'-CGCAG UUCCA-Ala
					**6%**	**7%**	**32**	**128**	**0.0364**	**0.00036**	5'**-CGCAG UUCCA-Ala-Ala**
16°C	5'-L-Ala-pCUG	1	5'-CGCAG	UUCCA	23%	27%	6.1	71	0.0470	0.00190	5'-CGCAG UUCCA-Ala
					**2%**	**2%**	**14**	**142**	**0.0246**	**0.00085**	5'**-CGCAG UUCCA-Ala-Ala**
	5'-L-Ala-pCUG	3	5'-CGCAG	UUCCA	43%	52%	4.8	74	0.0404	0.00239	5'-CGCAG UUCCA-Ala
					**7%**	**8%**	**14**	**139**	**0.0257**	**0.00081**	5'**-CGCAG UUCCA-Ala-Ala**
	5'-L-Ala-pCUG	5	5'-CGCAG	UUCCA	38%	46%	6.2	79	0.0410	0.00186	5'-CGCAG UUCCA-Ala
					**7%**	**8%**	**14**	**122**	**0.0306**	**0.00081**	5'**-CGCAG UUCCA-Ala-Ala**

Table S2. Kinetic data of two-step aminoacyl-transfer with 5'-CGCAGUUCCA. Conditions: 5'-CGCAGUUCCA 100 µM, HEPES 50 mM, NaCl 100 mM, $MgCl_2$ 5 mM, pH 6.8, 10°C.

Donor	Eq.	Acceptor		Obs. yield	Cor. yield	Diol ester half-life /h	Peak time min	k transfer /min^{-1}	k hydrolysis /min^{-1}	Notes
		Stem	Ovh							
5'-L-Ala-pCUGC	1	5'-CGCAG	UUCCA	48%	62%	6.6	63	0.0573	0.00175	one step only
5'-L-Ala-pCUGC	1	5'-CGCAG	UUCCA	36%	46%	3.3	/	/	0.00352	5'-CGCAG UUCCA-Ala
5'-F-Ala-pCUGCG	2			23%	/	99	618	0.0067	0.00012	5'-CGCAG UUCCA-Ala-Ala-F
				23%	/	137	668	0.0066	0.00008	5'-CGCAG UUCCA-Ala-F
5'-L-Ala-pCUGC	1	5'-CGCAG	UUCCA	32%	42%	1.9	/	/	0.00601	5'-CGCAG UUCCA-Ala
5'-F-Gly-pCUGCG	2			57%	/	80	411	0.0106	0.00014	5'-CGCAG UUCCA-Ala-Gly-F
				11%	/	107	686	0.0060	0.00011	5'-CGCAG UUCCA-Gly-F
5'-L-Leu-pCUGC	1	5'-CGCAG	UUCCA	27%	46%	14	97	0.0408	0.00083	one step only
5'-L-Leu-pCUGC	1	5'-CGCAG	UUCCA	16%	27%	2.6	/	/	0.00447	5'-CGCAG UUCCA-Leu
5'-F-Ala-pCUGCG	2			71%	/	159	715	0.0063	0.00007	5'-CGCAG UUCCA-Leu-Ala-F
5'-L-Leu-pCUGC	1	5'-CGCAG	UUCCA	16%	27%	1.2	/	/	0.00952	5'-CGCAG UUCCA-Leu
5'-F-Ala-pCUGCG	2			70%	/	129	382	0.0131	0.00009	5'-CGCAG UUCCA-Leu-Gly-F
5'-L-Pro-pCUGC	1	5'-CGCAG	UUCCA	26%	35%	1.8	50	0.0456	0.00631	one step only
5'-L-Pro-pCUGC	1	5'-CGCAG	UUCCA	31%	42%	1.3	/	/	0.00645	5'-CGCAG UUCCA-Pro
5'-F-Ala-pCUGCG	2			37%	/	36	218	0.0191	0.00032	5'-CGCAG UUCCA-Pro-Ala-F
5'-L-Pro-pCUGC	1	5'-CGCAG	UUCCA	26%	35%	0.2	/	/	0.06337	5'-CGCAG UUCCA-Pro
5'-F-Ala-pCUGCG	2			91%	/	10	48	0.0917	0.00117	5'-CGCAG UUCCA-Pro-Gly-F

Table S3. Kinetic data of two-step L-Ala- and fGly-transfer with 5'-CGCAGUUCCA and different donors. Conditions: 5'-CGCAGUUCCA 100 μM, HEPES 50 mM, NaCl 100 mM, $MgCl_2$ 5 mM, pH 6.8, 10°C.

Donor	Eq.	Acceptor		Obs. yield	Cor. yield	Diol ester half-life /h	Peak time	k transfer	k hydrolysis	Notes
		Stem	Ovh				min	/min^{-1}	/min^{-1}	
5'-L-Ala-pCUG	1	5'-CGCAG	UUCCA	34%	40%	1.0	/	/	0.01130	5'-CGCAG UUCCA-Ala
5'-F-Gly-pCUGCG	2			65%	/	74	325	0.0140	0.00016	5'-CGCAG UUCCA-Ala-Gly-F
5'-L-Ala-pCUGC	1	5'-CGCAG	UUCCA	32%	42%	1.9	/	/	0.00601	5'-CGCAG UUCCA-Ala
5'-F-Gly-pCUGCG	2			57%	/	82	410	0.0107	0.00014	5'-CGCAG UUCCA-Ala-Gly-F
5'-L-Ala-pCUGCG	1	5'-CGCAG	UUCCA	40%	49%	3.5	/	/	0.00330	5'-CGCAG UUCCA-Ala
5'-F-Gly-pCUGCG	2			40%	/	322	1002	0.0050	0.00004	5'-CGCAG UUCCA-Ala-Gly-F

Table S4. Oligonucleotides prepared and characterized for this study. F-, formyl-; Calc., calculated, theoretical value; Expt, experimental value, calculated from found value from LC-MS or observed raw number from MALDI-TOF; Found, raw number from LC-MS.

Sequence (5'-3')	Calc.	Expt.	Found
CGCAG UUCCA	3118.5	3118.5	1038.5
pCUG	974.13	973.9	972.9
pCUGC	1279.2	1279.0	638.5
pCUGCG	1624.2	1624.0	811.0
L-Ala-pCUG	1045.1	1044.9	1043.9
L-Ala-pCUGC	1350.2	1350.0	674.0
L-Ala-pCUGCG	1695.2	1693.4	845.7
L-Leu-pCUGCG	1737.3	1735.6	866.8
L-Pro-pCUGCG	1721.4	1720.8	859.4
F-Ala-pCUGCG	1723.3	1723.2	860.6
F-Gly-pCUGCG	1709.2	1708.8	853.4
CGCAG UUCCA-Ala	3189.5	3189.6	
CGCAG UUCCA-Ala-Ala	3260.6	3260.7	
CGCAG UUCCA-Ala-Ala-Ala	3331.7	3331.8	
CGCAG UUCCA-Ala-F	3217.5	3217.7	
CGCAG UUCCA-Ala-Ala-F	3288.6	3288.5	
CGCAG UUCCA-Gly-F	3204.5	3203.6	
CGCAG UUCCA-Ala-Gly-F	3274.6	3274.5	
CGCAG UUCCA-Leu-Ala-F	3330.6	3330.7	
CGCAG UUCCA-Leu-Gly-F	3316.6	3316.6	
CGCAG UUCCA-Pro-Ala-F	3314.6	3313.9	
CGCAG UUCCA-Pro-Gly-F	3300.6	3300.6	

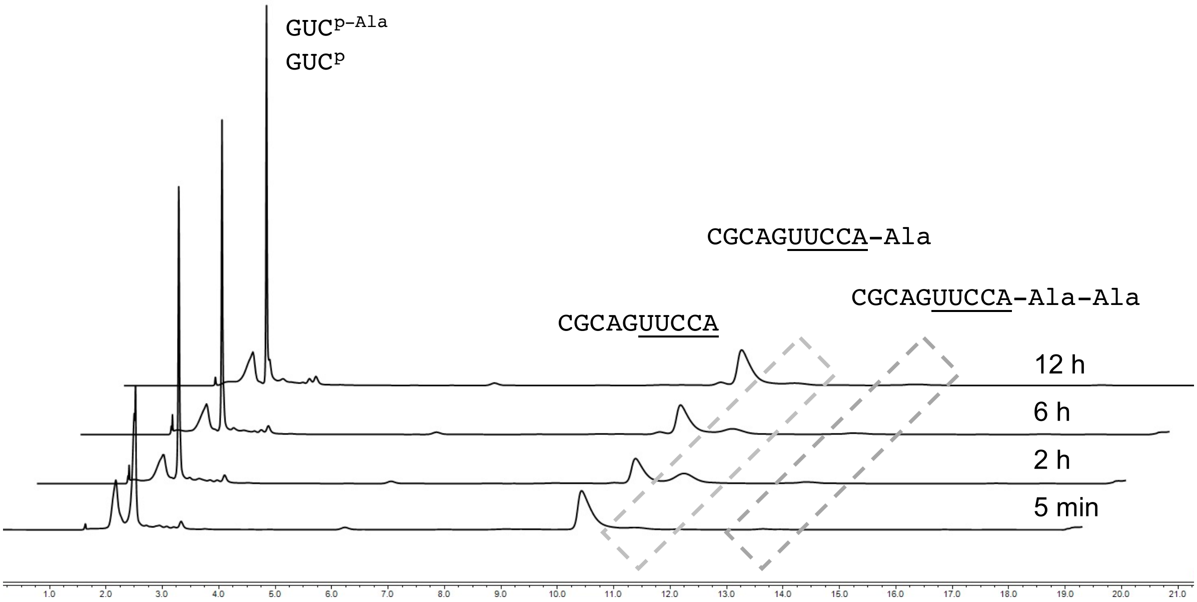

Figure S1. One-step L-Ala-transfer in a tRNA acceptor arm mimic. Stem-overhang sequence:

5'CGCAGUUCCA

3' GUCp-Ala …3 eq.

Transfer was monitored using HPLC with 260 nm UV detection. The solution was incubated at 16°C and aliquots of 8 µL were injected into an HPLC at different time points. Conditions: acceptor strand (100 µM), NaCl (100 mM), $MgCl_2$ (5 mM), HEPES (50 mM, pH 6.8).

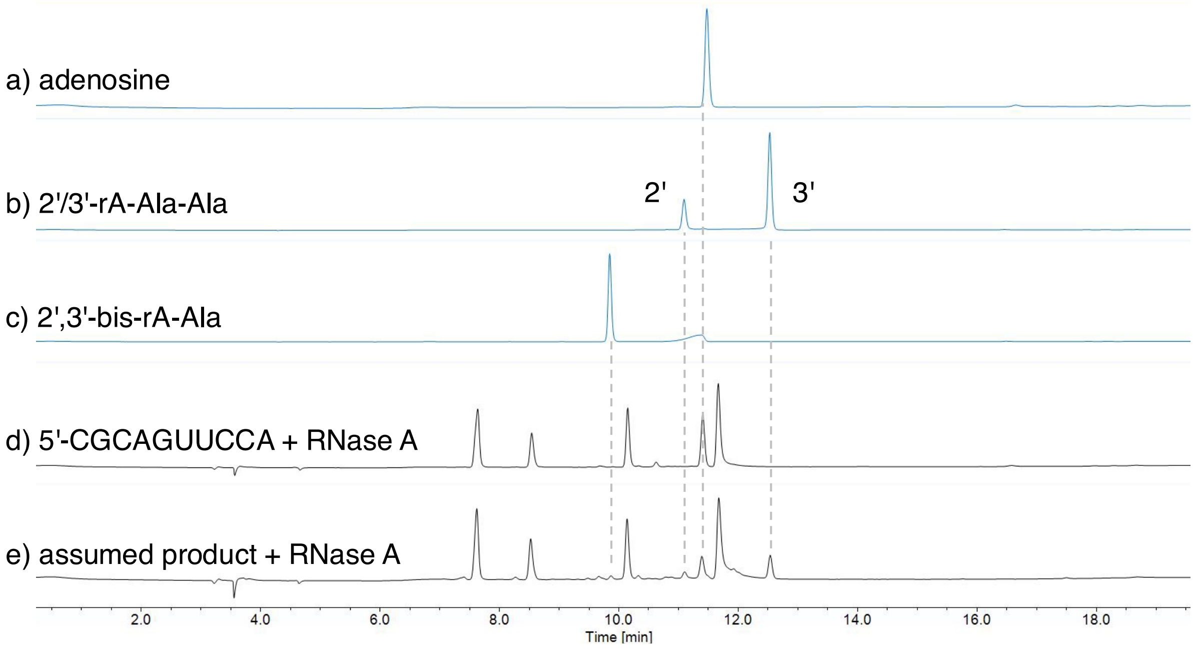

Figure S2. Enzymatic digestion confirms that the acceptor strand 3'-terminal adenosine is Ala-Ala-acylated. Peak assumed to be 5' CGCAGUUCCA-Ala-Ala was isolated, lyophilized and redissolved in water. An aliquot of 9 µL was added to 1 µL of sodium acetate solution buffer (NaOAc/HOAc, 0.5 M, pH 4.0). 0.2 µL of RNase A (10 mg/mL) was then added to the aliquot and the sample was then incubated at 20°C for 30 min. 10 µL of methanol was added, and the resulting mixture was centrifugated. The supernatant was analyzed by HPLC at 260 nm UV detection (e) by comparison with chromatograms of synthetic standards of adenosine (a) adenosine, (b) 2'/3'-Ala-Ala-adenosine, (c) bis-2',3'-Ala-adenosine, and (d) RNase A digested mixture of unacylated 5'-CGCAGUUCCA.

2',3'-bis-rA-Ala

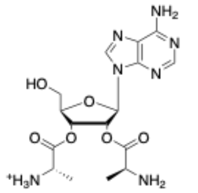

Calc. 410.18
Found 410.0

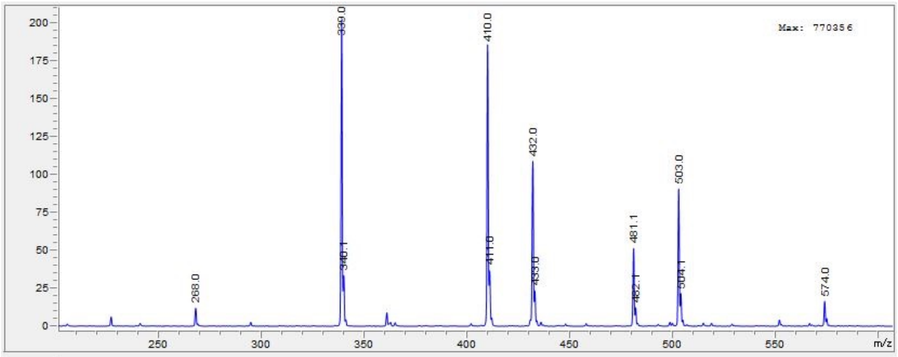

3'-rA-Ala-Ala

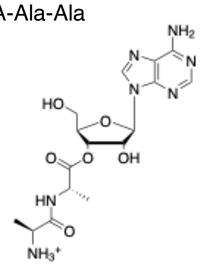

Calc. 410.18
Found 410.0

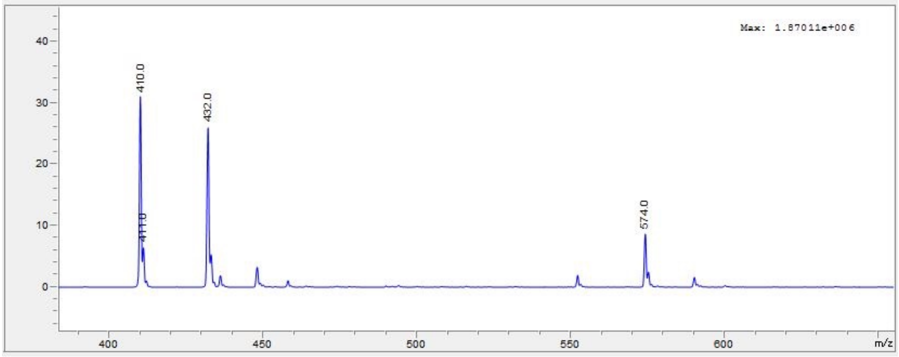

2'/3'-rA-Ala-Gly-F

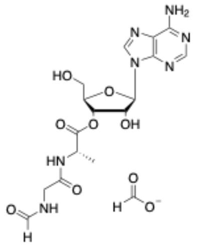

Calc. 468.39
Found 467.9

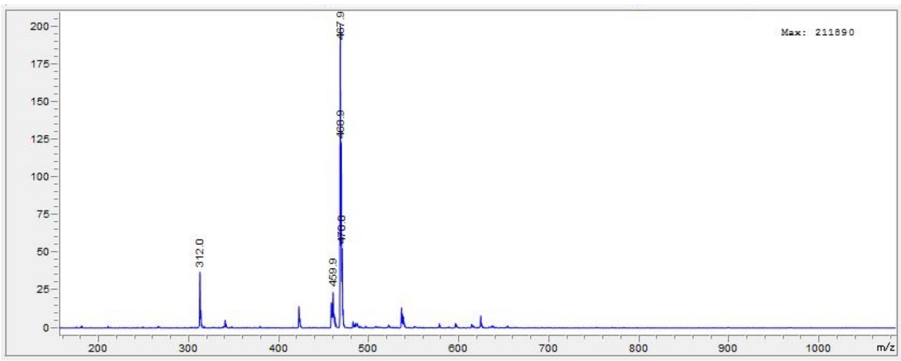

Figure S3. LCMS spectrum of the synthetic standards used to identify bis-2',3'-Ala-adenosine, 2'/3'-Ala-Ala-adenosine, and 2'/3'-fGly-Ala-adenosine.

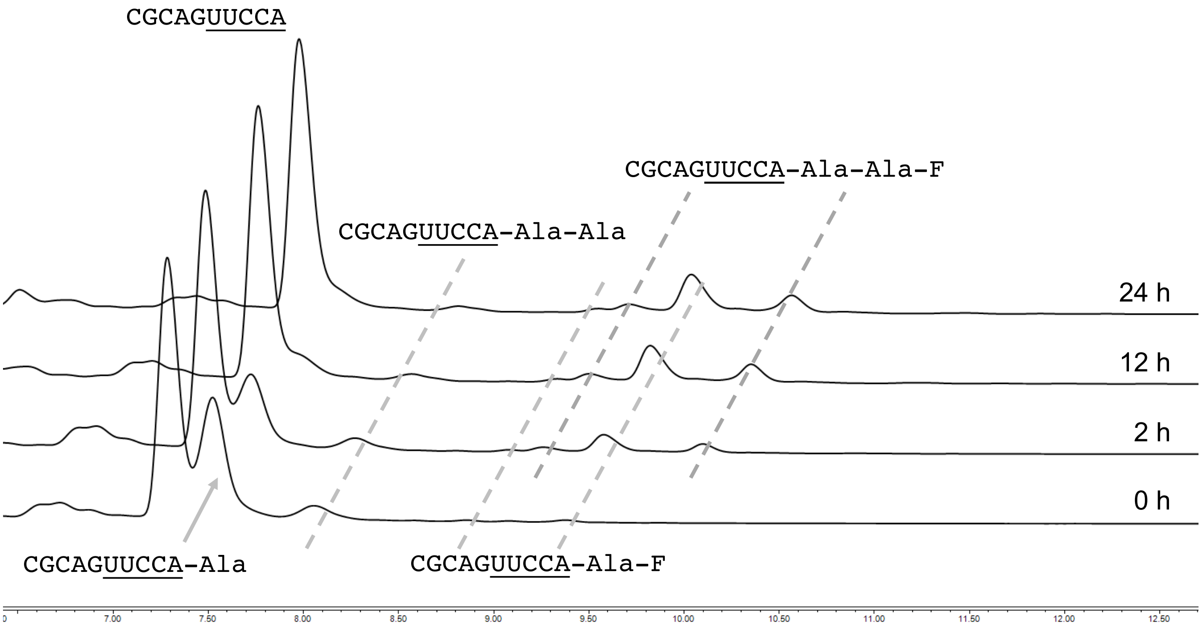

Figure S4. Two-step *N*-formyl-aminoacyl transfer in a tRNA acceptor arm mimic. Stem-overhang sequence:

5′CGCAGUUCCA

3′ CGUCp-Ala …step 1, 1 eq.

3′GCGUCp-Ala-F …step 2, 2 eq.

Transfer was monitored using HPLC with 260 nm UV detection. The solution was incubated at 10°C and aliquots of 8 μL were injected into an HPLC at different time points. Conditions: acceptor strand (100 μM), NaCl (100 mM), $MgCl_2$ (5 mM), HEPES (50 mM, pH 6.8). Ala-F, *N*-formyl-alanine.

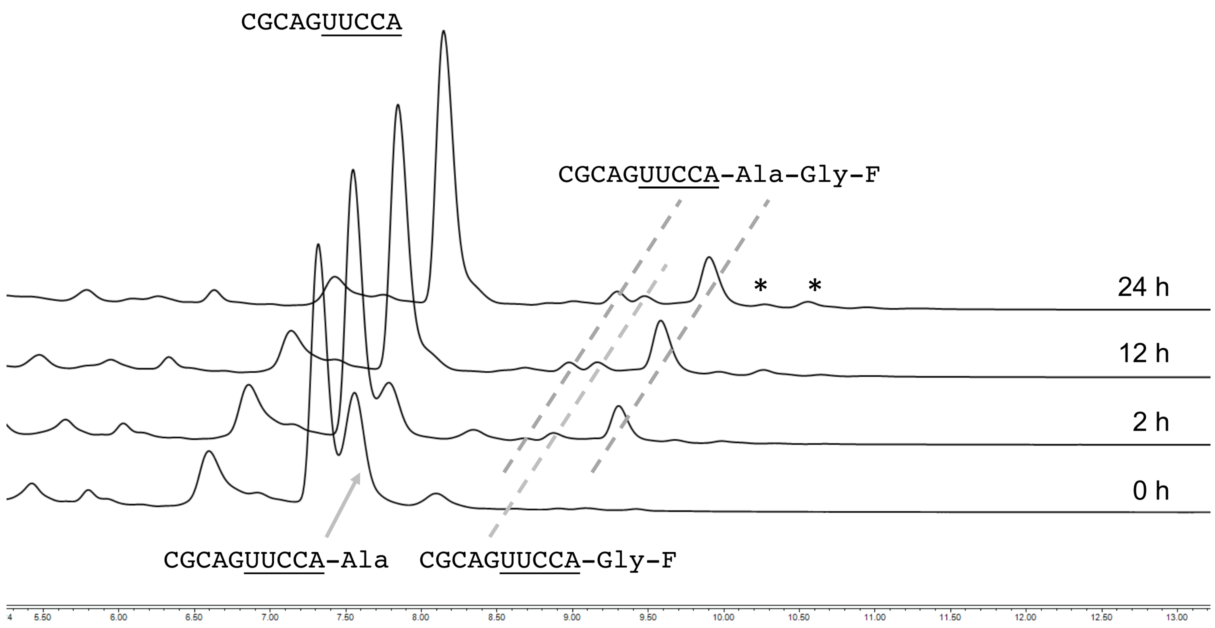

Figure S5. Two-step *N*-formyl-aminoacyl transfer in a tRNA acceptor arm mimic. Stem-overhang sequence:

```
5'CGCAGUUCCA
3' CGUCp-Ala     …step 1, 1 eq.
3'GCGUCp-Gly-F  …step 2, 2 eq.
```

Transfer was monitored using HPLC with 260 nm UV detection. The solution was incubated at 10°C and aliquots of 8 μL were injected into an HPLC at different time points. *, unidentified species. Conditions: acceptor strand (100 μM), NaCl (100 mM), $MgCl_2$ (5 mM), HEPES (50 mM, pH 6.8). Gly-F, *N*-formyl-glycine.

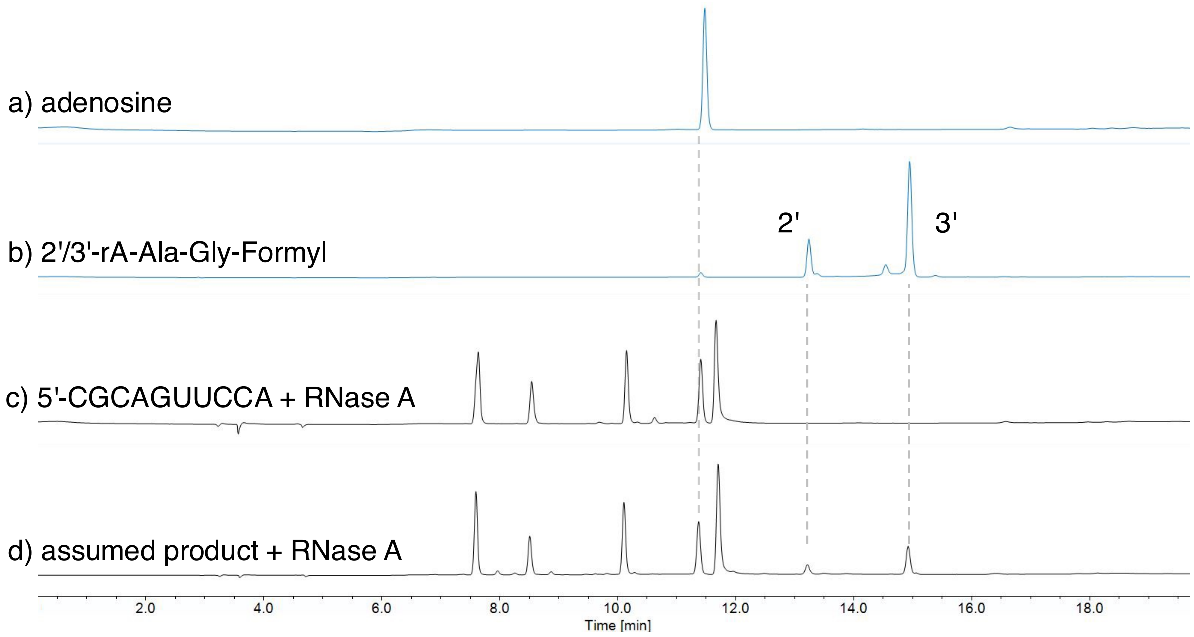

Figure S6. Enzymatic digestion confirms that the acceptor strand 3'-terminal adenosine is the 2'/3'-fGly-Ala ester. Peak assumed to be 5'CGCAGUUCCA-Ala-Gly-Formyl was isolated, lyophilized and redissolved in water. An aliquot of 9 µL was added to 1 µL of sodium acetate solution buffer (NaOAc/HOAc, 0.5 M, pH 4.0). 0.2 µL of RNase A (10 mg/mL) was added to the aliquot and the sample was then incubated at 20°C for 30 min. 10 µL of methanol was added, and the resulting mixture was centrifuged. The supernatant was analyzed by HPLC at 260 nm UV detection (d) by comparison with chromatograms of synthetic standards of (a) adenosine, (b) 2'/3'-fGly-Ala-adenosine, and (c) RNase A digested mixture of unacylated 5'-CGCAGUUCCA.

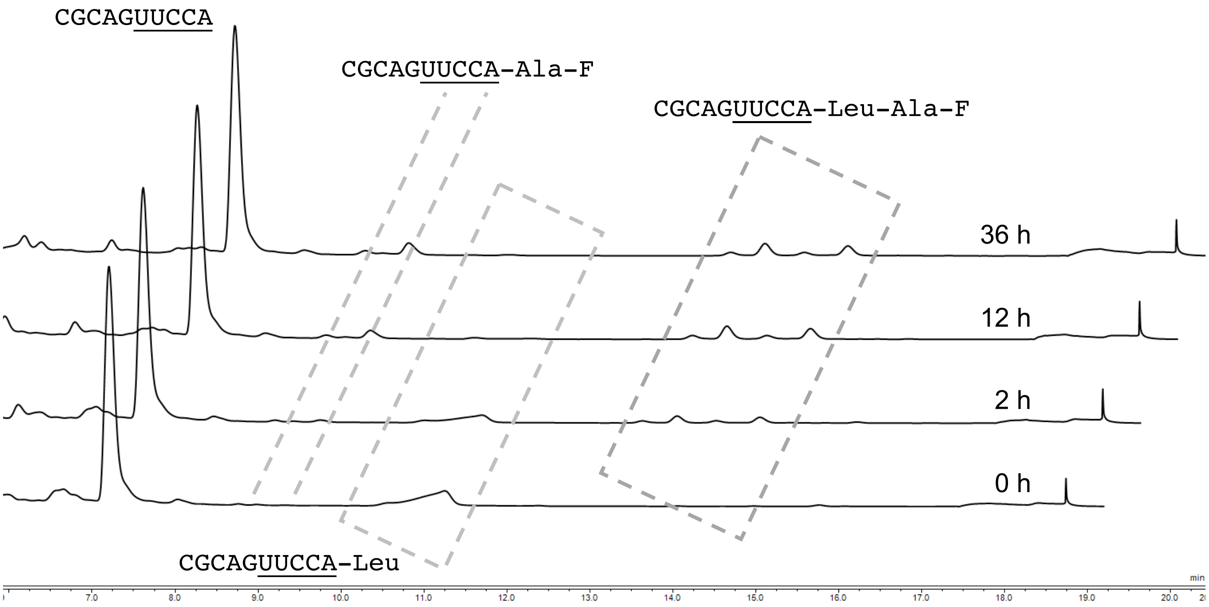

Figure S7. Two-step *N*-formyl-aminoacyl transfer in a tRNA acceptor arm mimic. Stem-overhang sequence:

5'CGCAGUUCCA

3' CGUCp-Leu …step 1, 1 eq.

3'GCGUCp-Ala-F …step 2, 2 eq.

Transfer was monitored using HPLC with 260 nm UV detection. The solution was incubated at 10°C and aliquots of 8 μL were injected into an HPLC at different time points. Conditions: acceptor strand (100 μM), NaCl (100 mM), $MgCl_2$ (5 mM), HEPES (50 mM, pH 6.8). Ala-F, *N*-formyl-alanine.

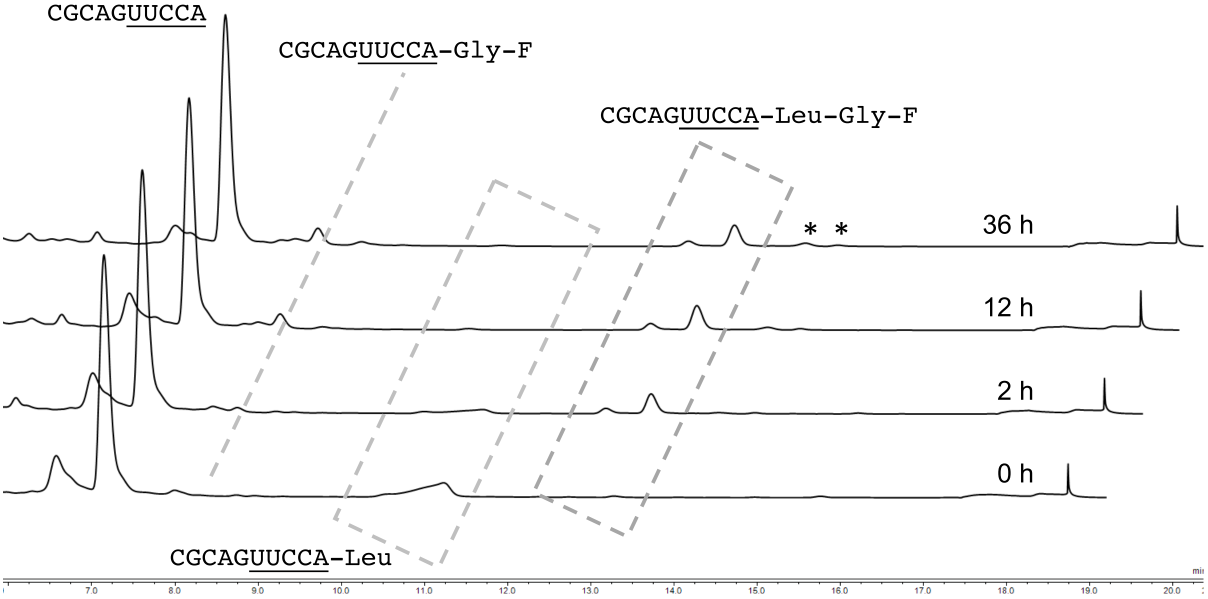

Figure S8. Two-step *N*-formyl-aminoacyl transfer in a tRNA acceptor arm mimic. Stem-overhang sequence:

`5'CGCAGUUCCA`

`3' CGUCp-Leu` …step 1, 1 eq.

`3'GCGUCp-Gly-F` …step 2, 2 eq.

Transfer was monitored using HPLC with 260 nm UV detection. The solution was incubated at 10°C and aliquots of 8 µL were injected into an HPLC at different time points. *, unidentified species. Conditions: acceptor strand (100 µM), NaCl (100 mM), $MgCl_2$ (5 mM), HEPES (50 mM, pH 6.8). Gly-F, *N*-formyl-glycine.

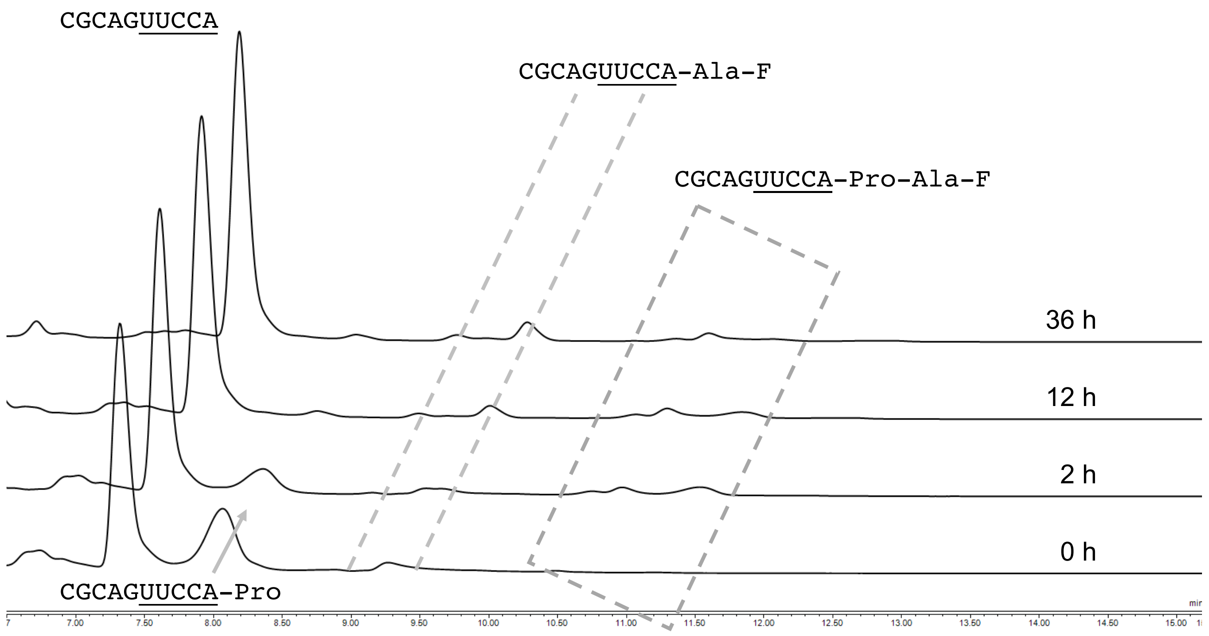

Figure S9. Two-steps *N*-formyl-aminoacyl transfer in a tRNA acceptor arm mimic. Stem-overhang sequence:

`5'CGCAGUUCCA`

`3' CGUCp-Pro` …step 1, 1 eq.

`3'GCGUCp-Ala-F` …step 2, 2 eq.

Transfer was monitored using HPLC with 260 nm UV detection. The solution was incubated at 10°C and aliquots of 8 µL were injected into an HPLC at different time points. Conditions: acceptor strand (100 µM), NaCl (100 mM), $MgCl_2$ (5 mM), HEPES (50 mM, pH 6.8). Ala-F, *N*-formyl-alanine.

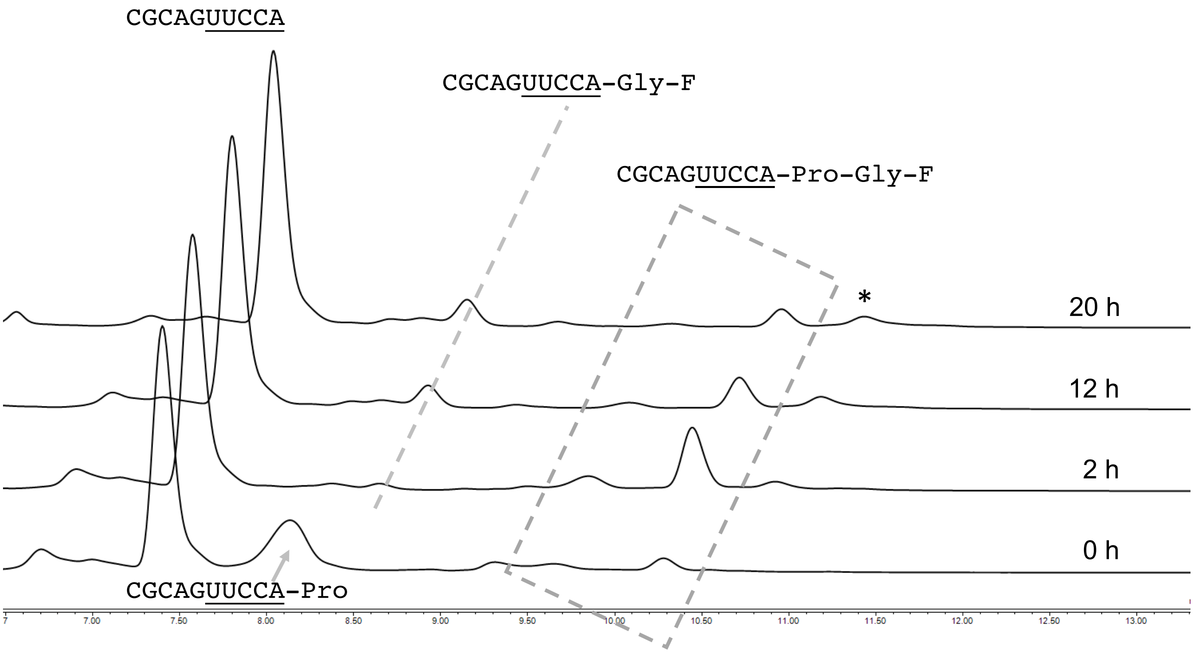

Figure S10. Two-step *N*-formyl-aminoacyl transfer in a tRNA acceptor arm mimic. Stem-overhang sequence:

5′CGCAGUUCCA

3′ CGUCp-Pro …step 1, 1 eq.

3′GCGUCp-Gly-F …step 2, 2 eq.

Transfer was monitored using HPLC with 260 nm UV detection. The solution was incubated at 10°C and aliquots of 8 µL were injected into an HPLC at different time points. *, unidentified specie. Conditions: acceptor strand (100 µM), NaCl (100 mM), $MgCl_2$ (5 mM), HEPES (50 mM, pH 6.8). Gly-F, *N*-formyl-glycine.

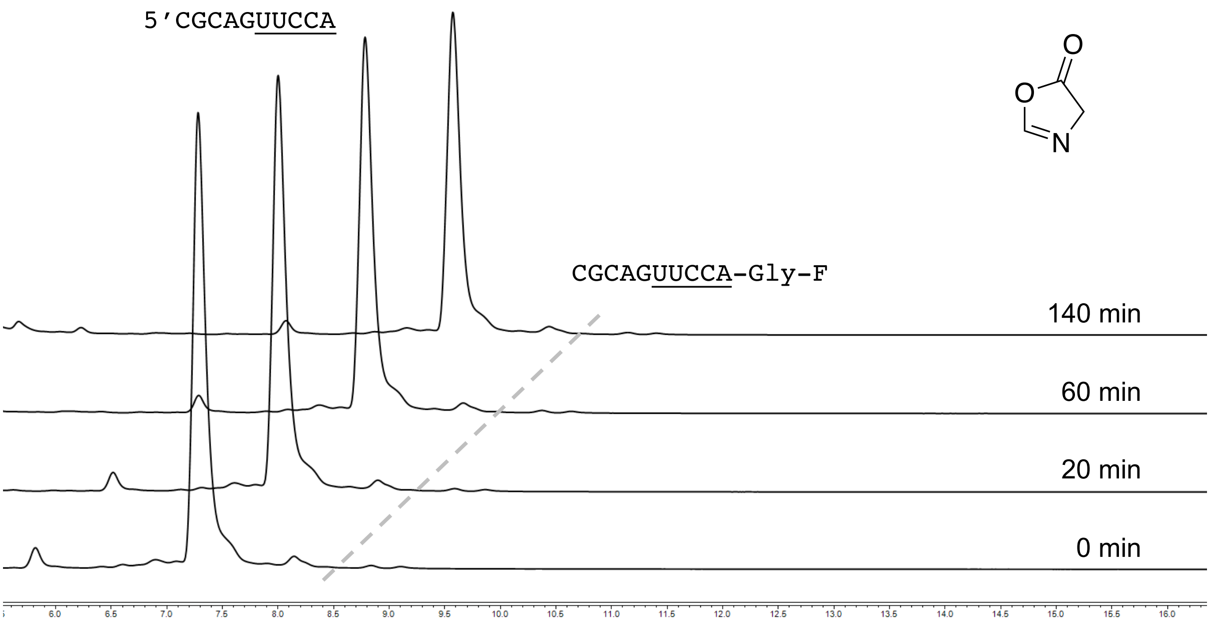

Figure S11. Attempted one-step *N*-formyl-aminoacyl transfer from *in situ* formed glycine 5-(*4H*)-oxazolone (inset) in a tRNA acceptor arm mimic. Stem-overhang sequence:

5′CGCAGUUCCA

3′GCGUCp ...1 eq.

Transfer was monitored using HPLC with 260 nm UV detection. The solution was incubated at 10°C and aliquots of 8 µL were injected into an HPLC at different time points. Conditions: acceptor strand (100 µM), donor strand (100 µM), NaCl (100 mM), $MgCl_2$ (5 mM), HEPES (50 mM, pH 6.8), formyl-glycine (10 mM), EDC HCl (60 mM). An additional aliquot of EDC (60 mM) was added after 1 h. Gly-F, *N*-formyl-glycine.

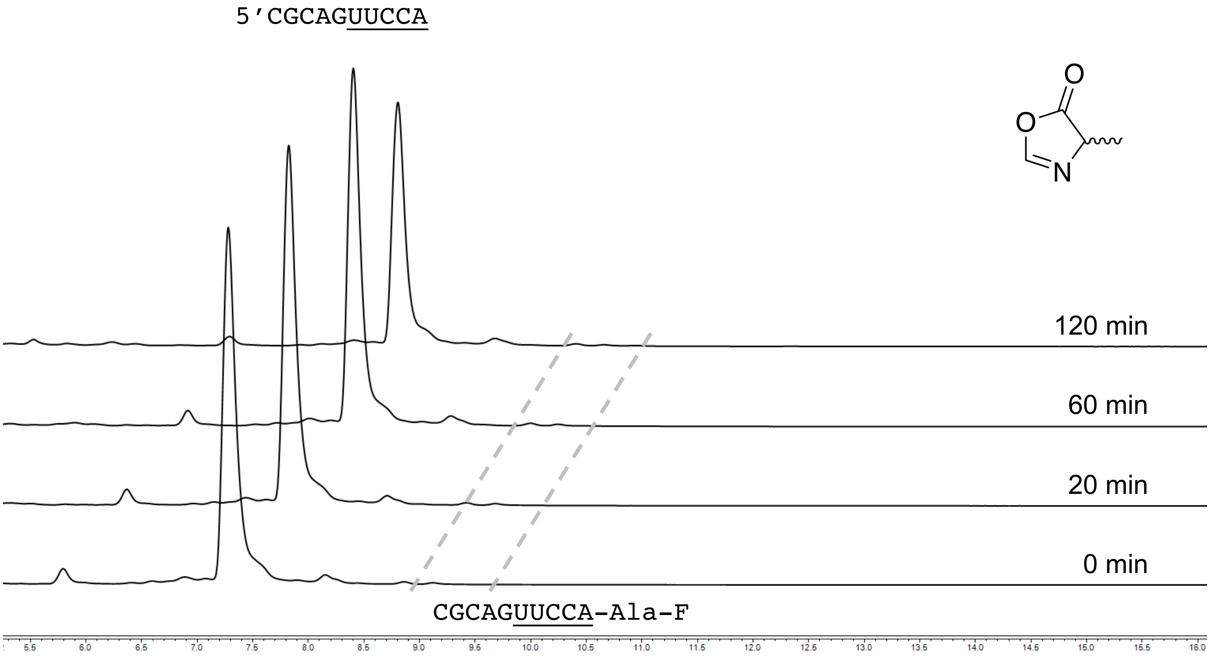

Figure S12. Attempted one-step *N*-formyl-aminoacyl transfer from *in situ* formed alanine 5-(*4H*)-oxazolone (inset) in a tRNA acceptor arm mimic. Stem-overhang sequence:

5'CGCAGUUCCA

3'GCGUCp ...1 eq.

Transfer was monitored using HPLC with 260 nm UV detection. The solution was incubated at 10°C and aliquots of 8 µL were injected into an HPLC at different time points. Conditions: acceptor strand (100 µM), NaCl (100 mM), $MgCl_2$ (5 mM), HEPES (50 mM, pH 6.8), formyl-alanine (10 mM), EDC HCl (60 mM). An additional aliquot of EDC (60 mM) was added after 1 h. Ala-F, *N*-formyl-alanine.

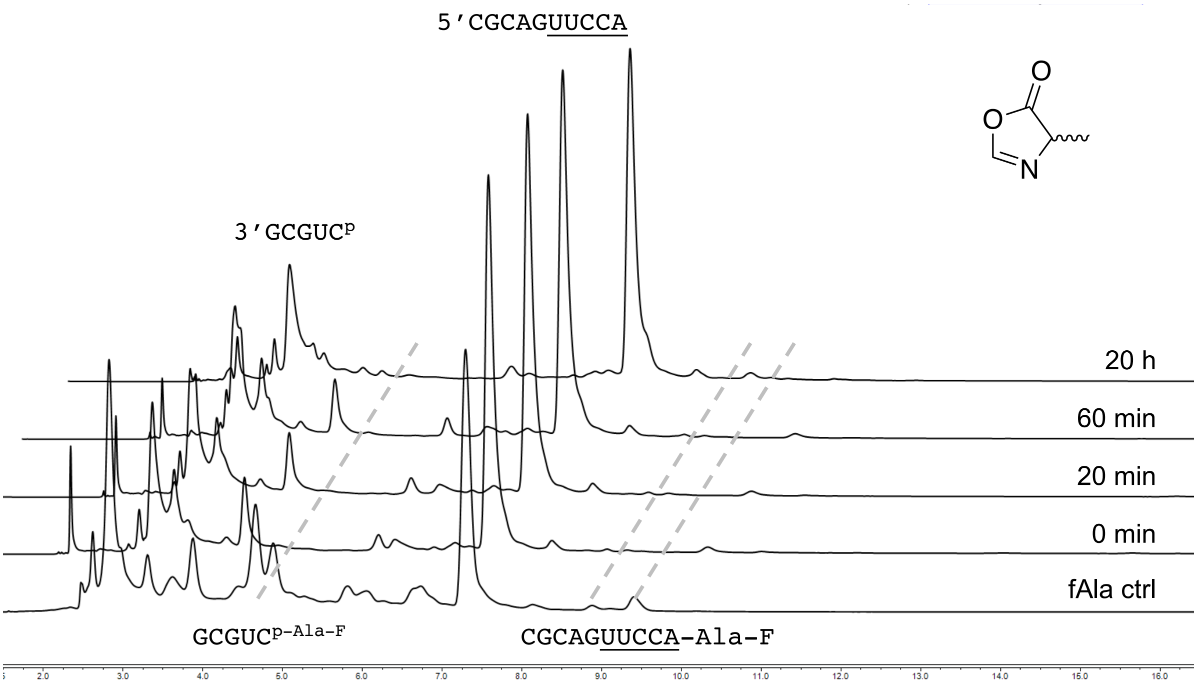

Figure S13. Attempted one-step *N*-formyl-aminoacyl transfer from alanine 5-(*4H*)-oxazolone (inset) in a tRNA acceptor arm mimic. Stem-overhang sequence:

5′CGCAGUUCCA

3′GCGUCp ... 1 eq. + alanine 5-(*4H*)-oxazolone … 2000 eq.

Transfer was monitored using HPLC with 260 nm UV detection. The solution was incubated at 10°C and aliquots of 8 µL were injected into an HPLC at different time points. Conditions: acceptor strand (100 µM), donor strand (100 µM), NaCl (100 mM), $MgCl_2$ (5 mM), HEPES (50 mM, pH 6.8), Ala 5-(*4H*)-oxazolone (200 mM), 5% MeCN. Ala-F, *N*-formyl-alanine.

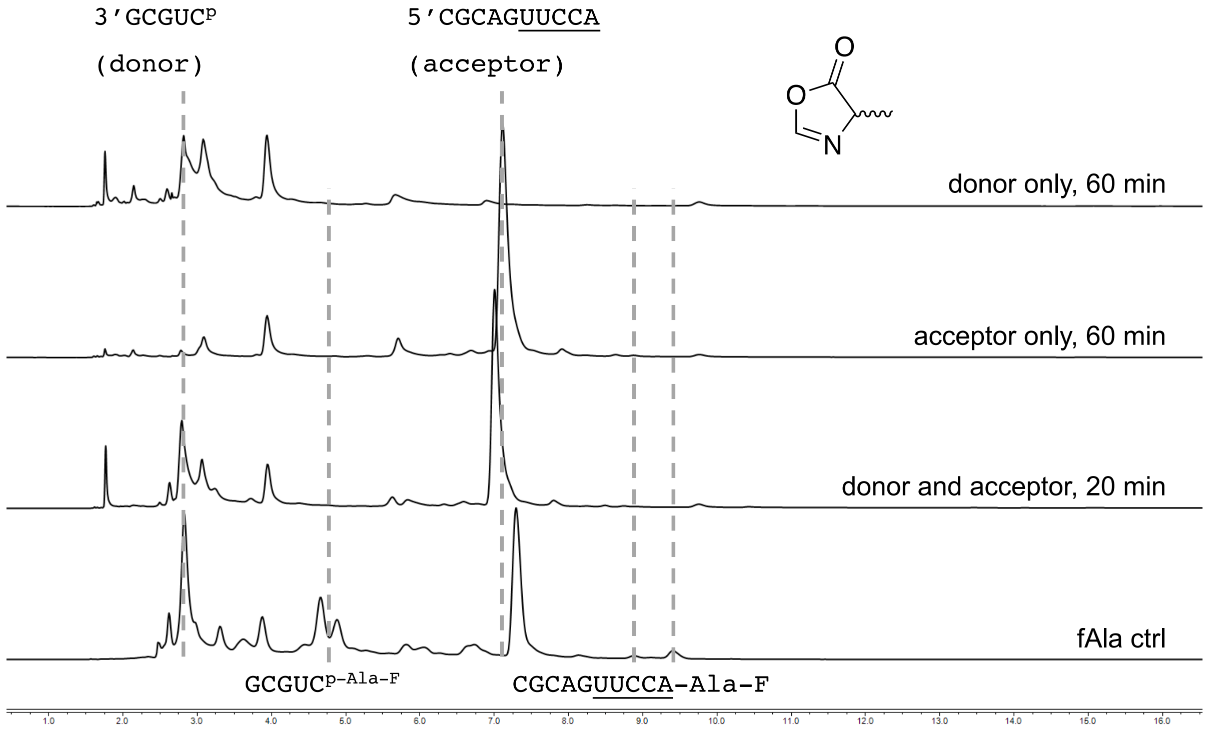

Figure S14. Controls for attempted one-step *N*-formyl-aminoacyl transfer from alanine 5-(*4H*)-oxazolone (inset) with donor and/or acceptor strand. Sequence:

5'CGCAGUUCCA

and / or

5'pCUGCG

Reactions were monitored using HPLC with 260 nm UV detection. The solution was incubated at 10°C and aliquots of 8 µL were injected into an HPLC at different time points. Conditions: RNA (100 µM), NaCl (100 mM), $MgCl_2$ (5 mM), HEPES (50 mM, pH 6.8), alanine 5-(*4H*)-oxazolone (200 mM), 5% MeCN. Ala-F, *N*-formyl-alanine.

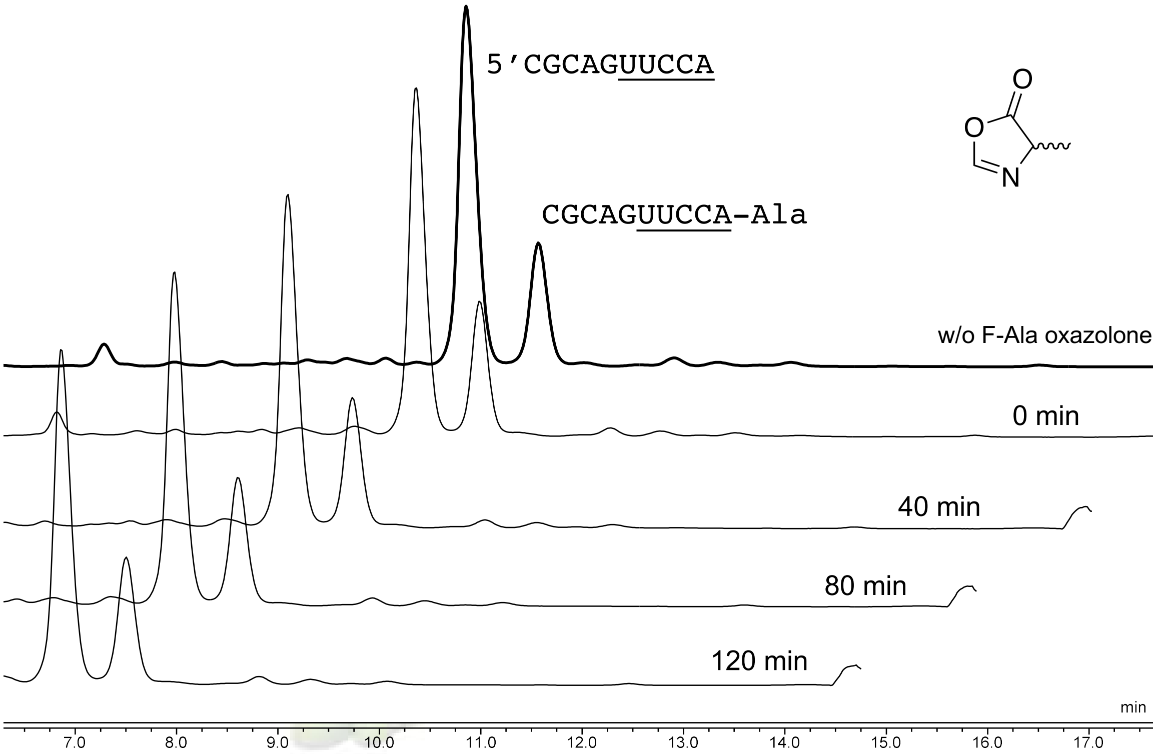

Figure S15. Attempted two-step *N*-formyl-aminoacyl transfer from alanine 5-(*4H*)-oxazolone (inset) in an alanyl-tRNA acceptor arm mimic. Stem-overhang sequence:

`5'CGCAGUUCCA`

`3' CGUCp-Ala` …step 1, 1 eq.

Transfer was monitored using HPLC with 260 nm UV detection. The solution was incubated at 10°C and aliquots of 6 µL were injected into an HPLC at different time points. Conditions: acceptor strand (100 µM), donor strand (100 µM), NaCl (100 mM), $MgCl_2$ (5 mM), HEPES (50 mM, pH 6.8); then Ala 5-(*4H*)-oxazolone (200 mM), 5% MeCN.

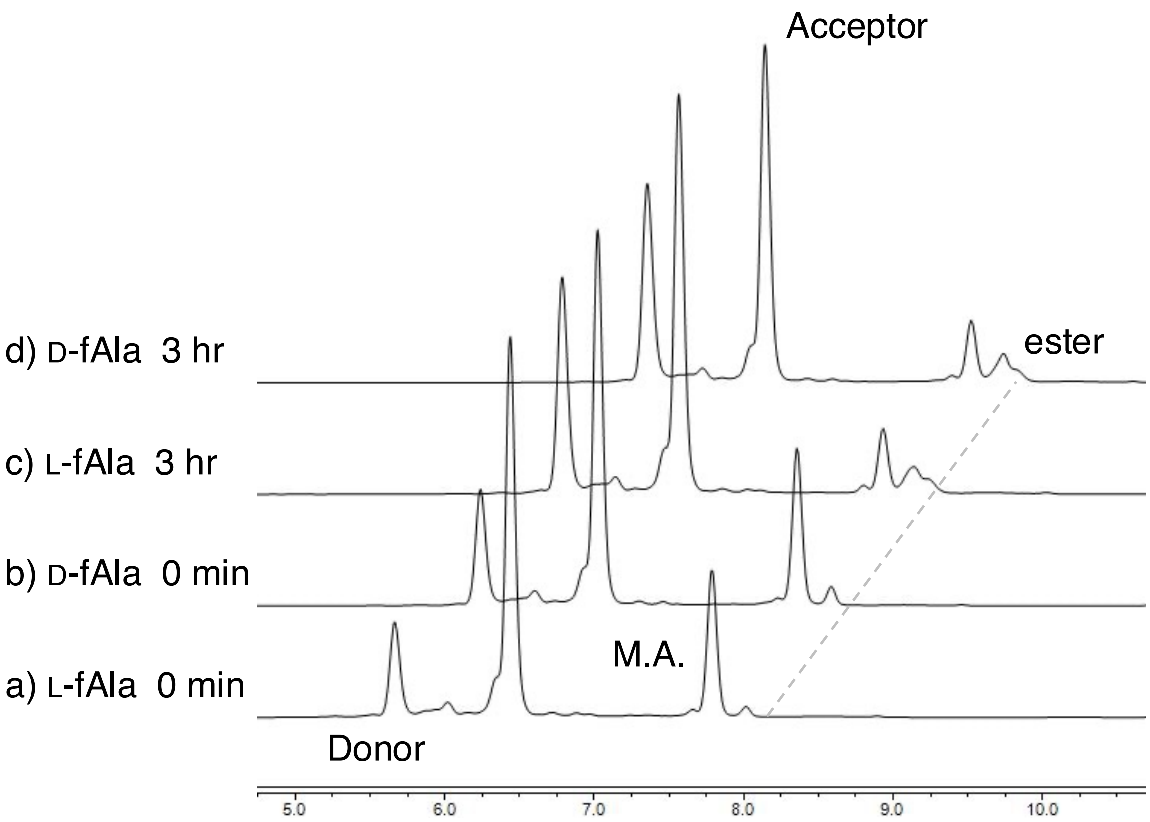

Figure S16. One-step *N*-formyl-alanyl transfer in a tRNA acceptor arm mimic. Stem-overhang sequence:

`5'UCGCUUUCCA`

`3'AGCGAp-Ala-F` 1 eq.

Transfer was monitored using HPLC with 260 nm UV detection. The solution was incubated at 10°C and aliquots of 5 μL were injected into an HPLC at different time points. Conditions: acceptor strand (100 μM), NaCl (100 mM), $MgCl_2$ (16.6 mM), HEPES (50 mM, pH 8.0). Ala-F, *N*-formylalanine. Transfer from a mixed anhydride formed from a) L-*N*-formyl-alanine or b) D-*N*-formyl-alanine at 0 min. Transfer from a mixed anhydride formed from c) L-*N*-formyl-alanine or d) D-*N*-formyl-alanine at 3 hr. M.A., mixed anhydride.

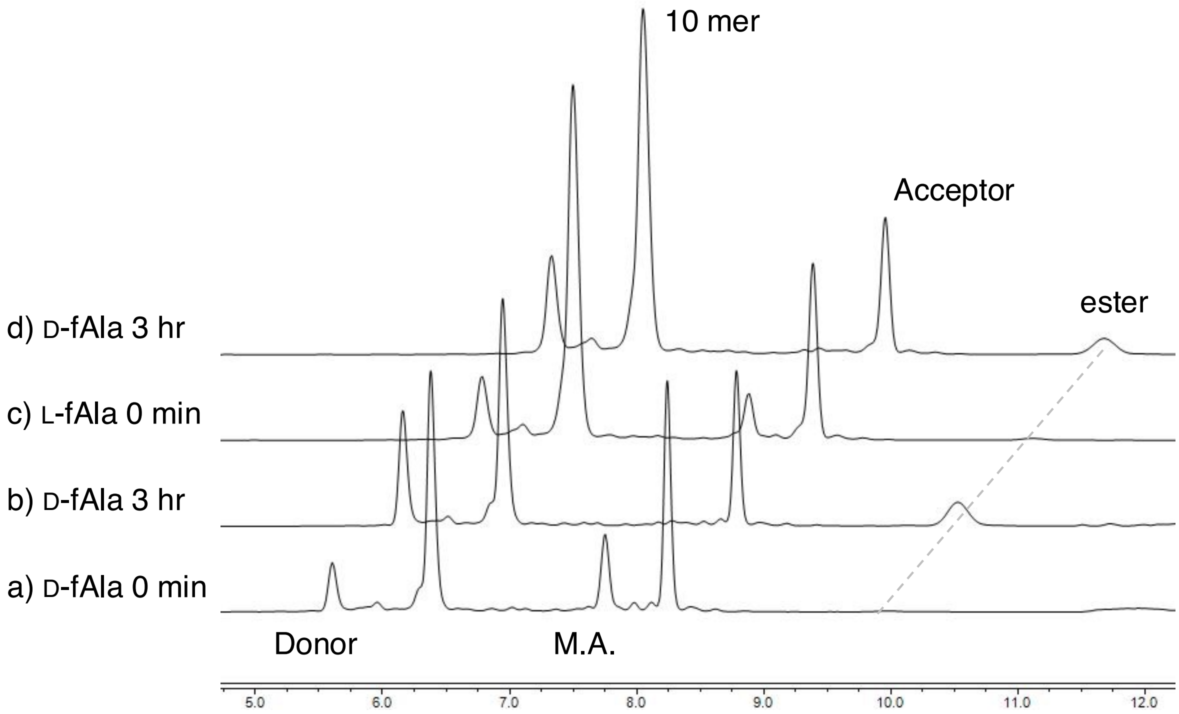

Figure S17. One-step *N*-formyl-alanyl transfer in a nicked duplex. Nicked duplex sequence:

5′UCGCUUUCCA

3′AGCGAp-Ala-F 1 eq.

3′AAGGUAAU (acceptor)

Transfer was monitored using HPLC with 260 nm UV detection. The solution was incubated at 10°C and aliquots of 5 μL were injected into an HPLC at different time points. Conditions: acceptor strand (100 μM), template strand (100 μM), NaCl (100 mM), $MgCl_2$ (16.6 mM), HEPES (50 mM, pH 8). Ala-F, *N*-formylalanine. Transfer from a mixed anhydride formed from D-*N*-formyl-alanine at a) 0 min; b) 3 hr. Transfer from a mixed anhydride formed from L-*N*-formyl-alanine at c) 0 min; d) 3 hr. M.A., mixed anhydride.

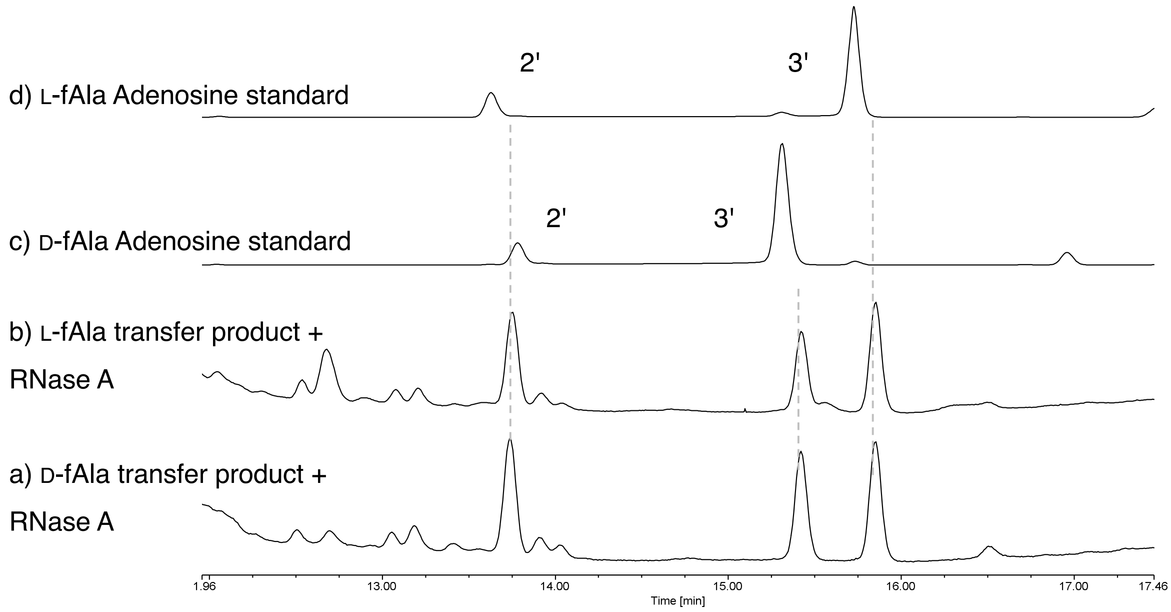

Figure S18. Enzymatic digestion confirms both L- and D-*N*-fAla residues transfer across a nicked loop (from Fig. S16). A 9 µL aliquot of the crude products from the transfer of L- or D-*N*-fAla mixed anhydride 5mer onto an acceptor 10mer was added to 1 µL of sodium acetate solution buffer (NaOAc/HOAc, 0.5 M, pH 4.0). 1 µL of RNase A (10 mg/mL) was then added to the aliquot and the sample was then incubated at 20°C for 30 min. 11 µL of methanol was added, and the resulting mixture was centrifugated. The supernatant from the transfer and digestion of a) D- and b) L-*N*-fAla mixed anhydride was analyzed by HPLC at 260 nm UV detection and compared to the c) D- and d) L-2'/3'-*N*-fAla-adenosine ester standards.

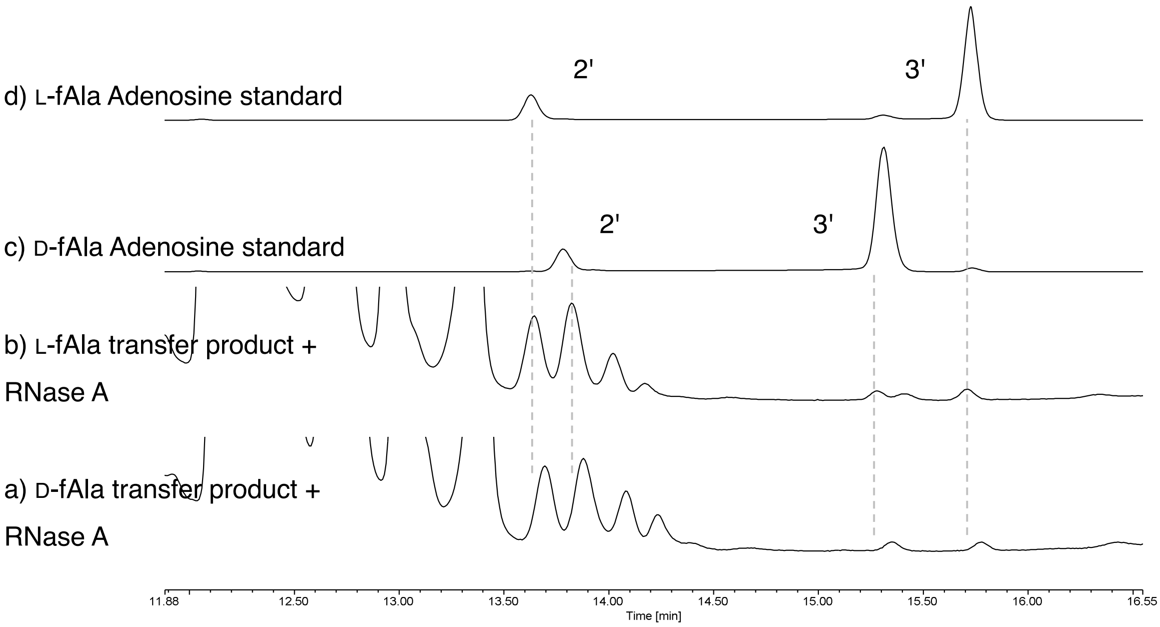

Figure S19. Enzymatic digestion confirms both L- and D-*N*-fAla residues transfer across a nicked duplex (from Fig. S17). A 9 µL aliquot of the crude products from the transfer of L- or D-*N*-fAla mixed anhydride 5mer onto an acceptor 8mer was added to 1 µL of sodium acetate solution buffer (NaOAc/HOAc, 0.5 M, pH 4.0). 1 µL of RNase A (10 mg/mL) was then added to the aliquot and the sample was then incubated at 20°C for 30 min. 11 µL of methanol was added, and the resulting mixture was centrifugated. The supernatant from the transfer and digestion of a) D- and b) L-*N*-fAla mixed anhydride was analyzed by HPLC at 260 nm UV detection and compared to the c) D- and d) L-2'/3'-*N*-fAla-adenosine ester standards.

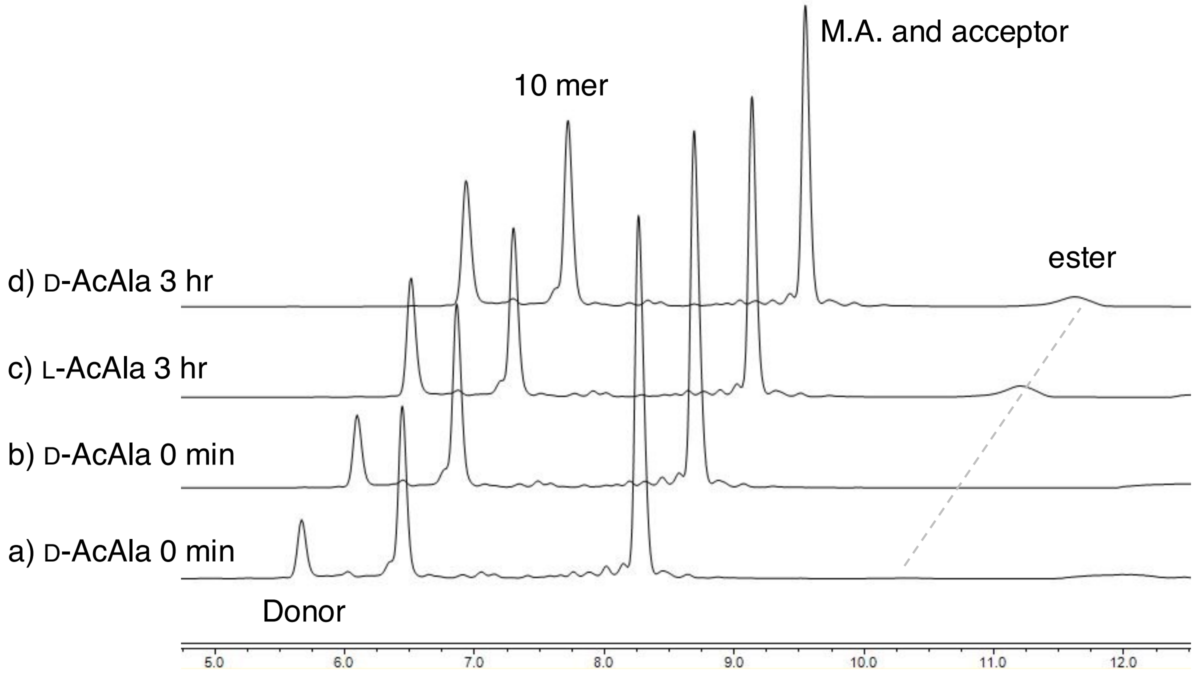

Figure S20. One-step *N*-acetyl-alanyl transfer in a nicked duplex. Nicked duplex sequence:

`5'UCGCUUUCCA`

`3'AGCGAp-Ala-Ac 1` eq.

`3'AAGGUAAU`

Transfer was monitored using HPLC with 260 nm UV detection. The solution was incubated at 10°C and aliquots of 5 µL were injected into an HPLC at different time points. Conditions: acceptor strand (100 µM), template strand (100 µM), NaCl (100 mM), $MgCl_2$ (16.6 mM), HEPES (50 mM, pH 8). Ala-Ac, *N*-acetyl-alanine. Transfer from a mixed anhydride formed from a) L-*N*-acetyl-alanine or b) D-*N*-acetyl-alanine at 0 min. Transfer from a mixed anhydride formed from c) L-*N*-acetyl-alanine or d) D-*N*-acetyl-alanine at 3 hr. M.A., mixed anhydride.

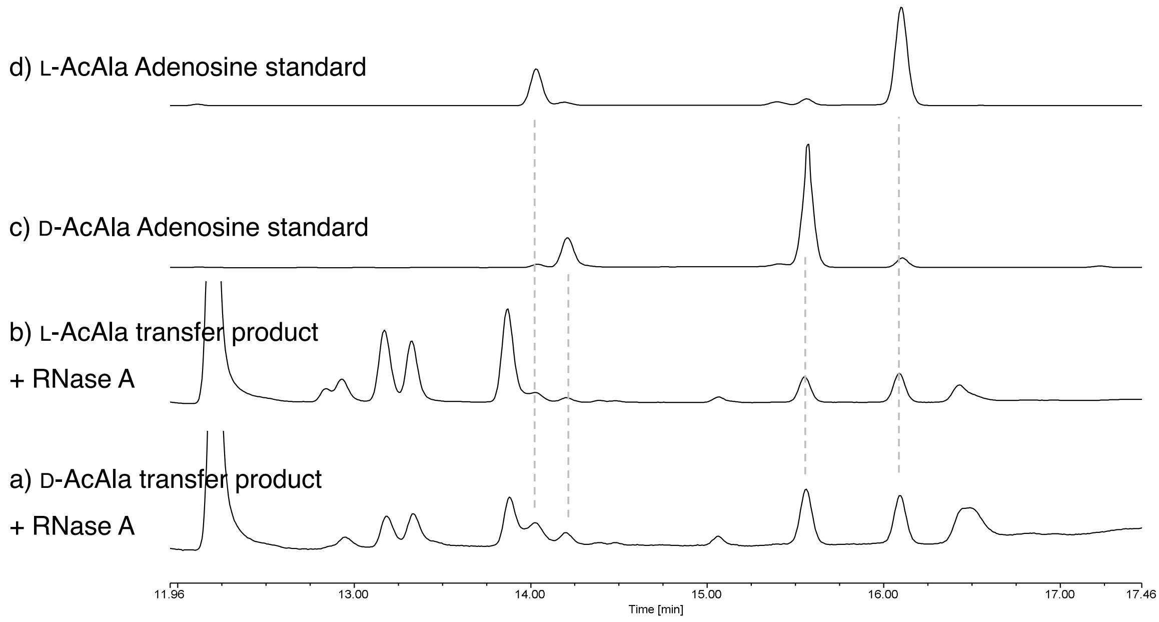

Figure S21. Enzymatic digestion confirms both L- and D-*N*-AcAla residues transfer across a nicked duplex (from Fig. S20). The peak corresponding to 5'-UAAUGGAA-AcAla was isolated, lyophilized and redissolved in water. An aliquot of 9 μL was added to 1 μL of sodium acetate solution buffer (NaOAc/HOAc, 0.5 M, pH 4.0). 1 μL of RNase A (10 mg/mL) was then added to the aliquot and the sample was then incubated at 20°C for 30 min. 11 μL of methanol was added, and the resulting mixture was centrifugated. The supernatant from the transfer and digestion of a) D- and b) L-*N*-AcAla mixed anhydride was analyzed by HPLC at 260 nm UV detection and compared to the c) D- and d) L-2'/3'-*N*-AcAla-adenosine ester standards.

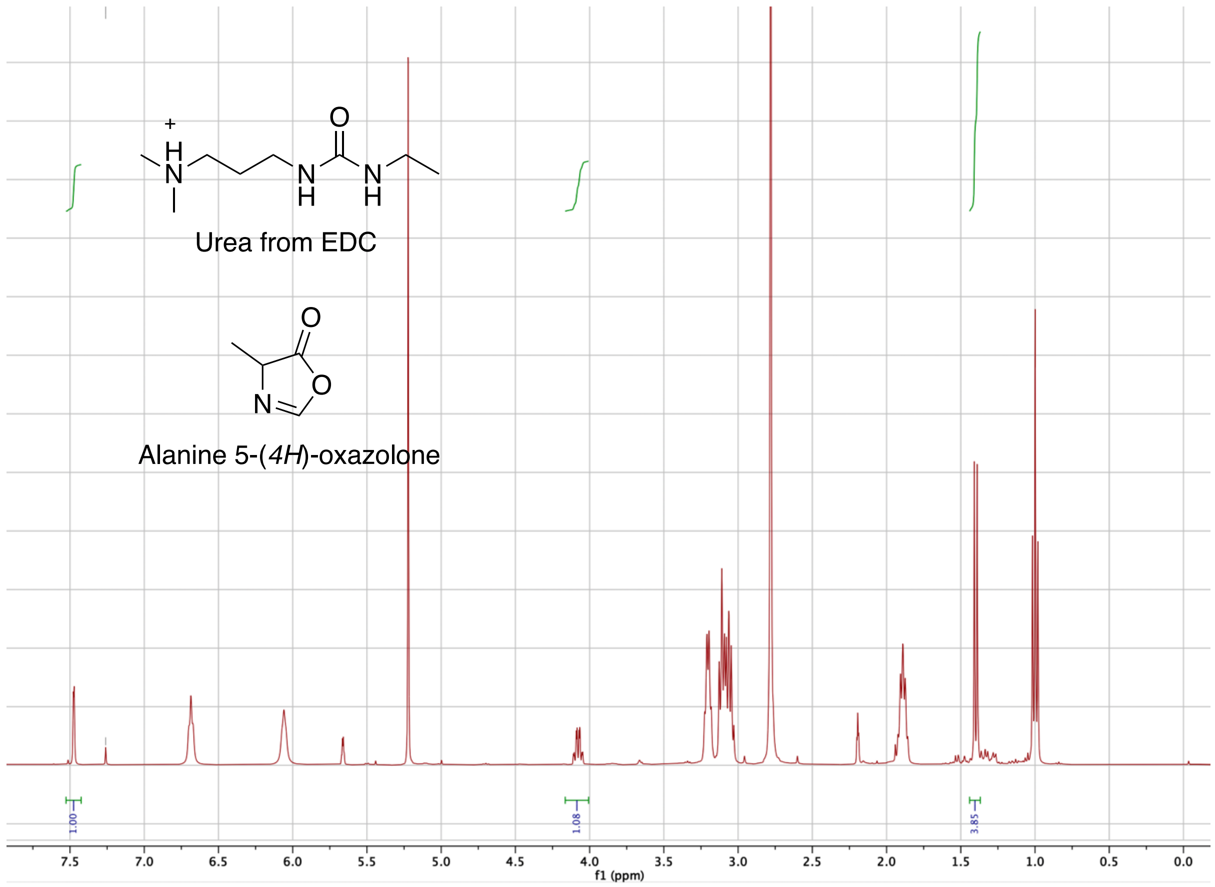

Figure S22. ^{1}H-NMR spectrum of crude product of alanine 5-(*4H*)-oxazolone. Integrated peaks are those corresponding to alanine 5-(*4H*)-oxazolone.

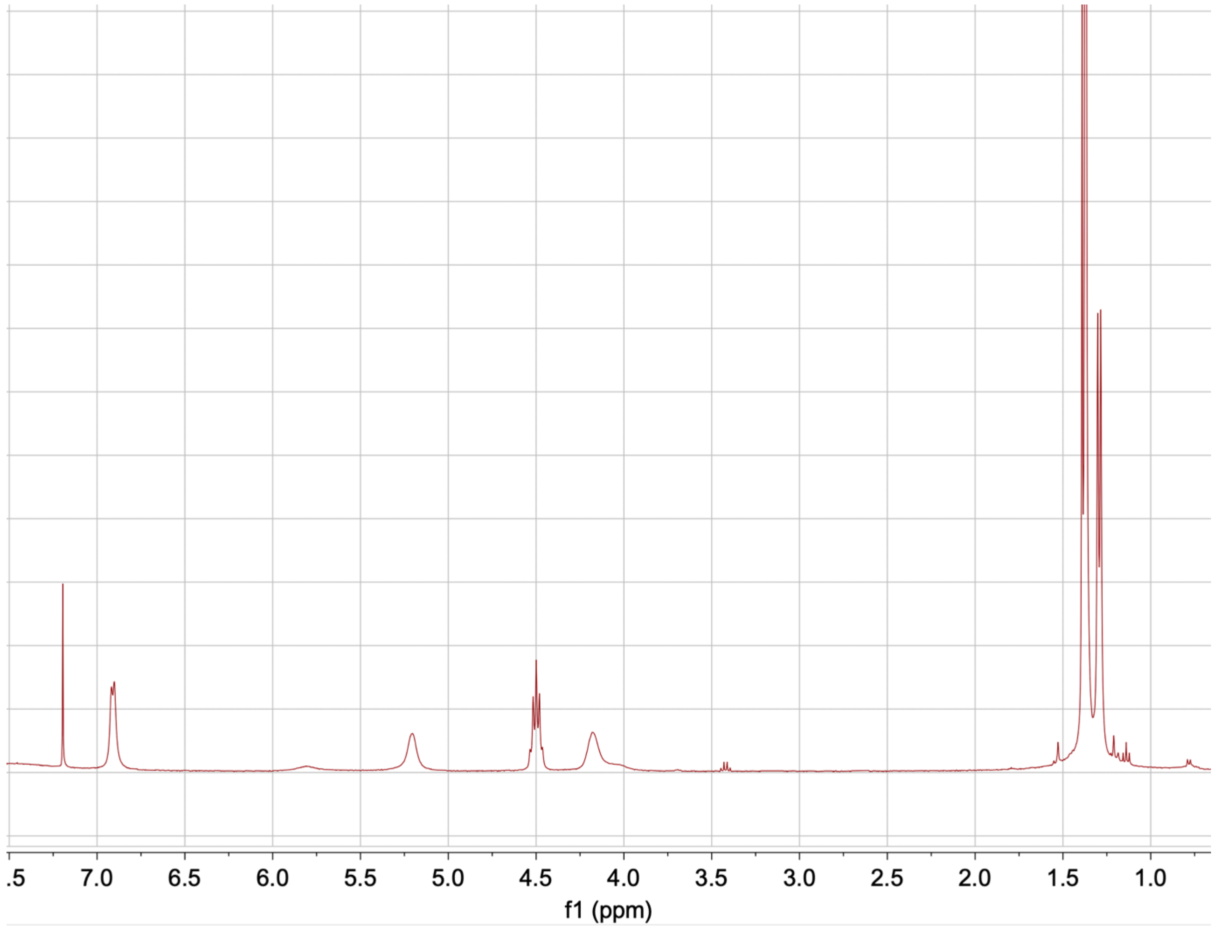

Figure S23. ^{1}H-NMR spectrum of *N*-Boc-alanylalanine.

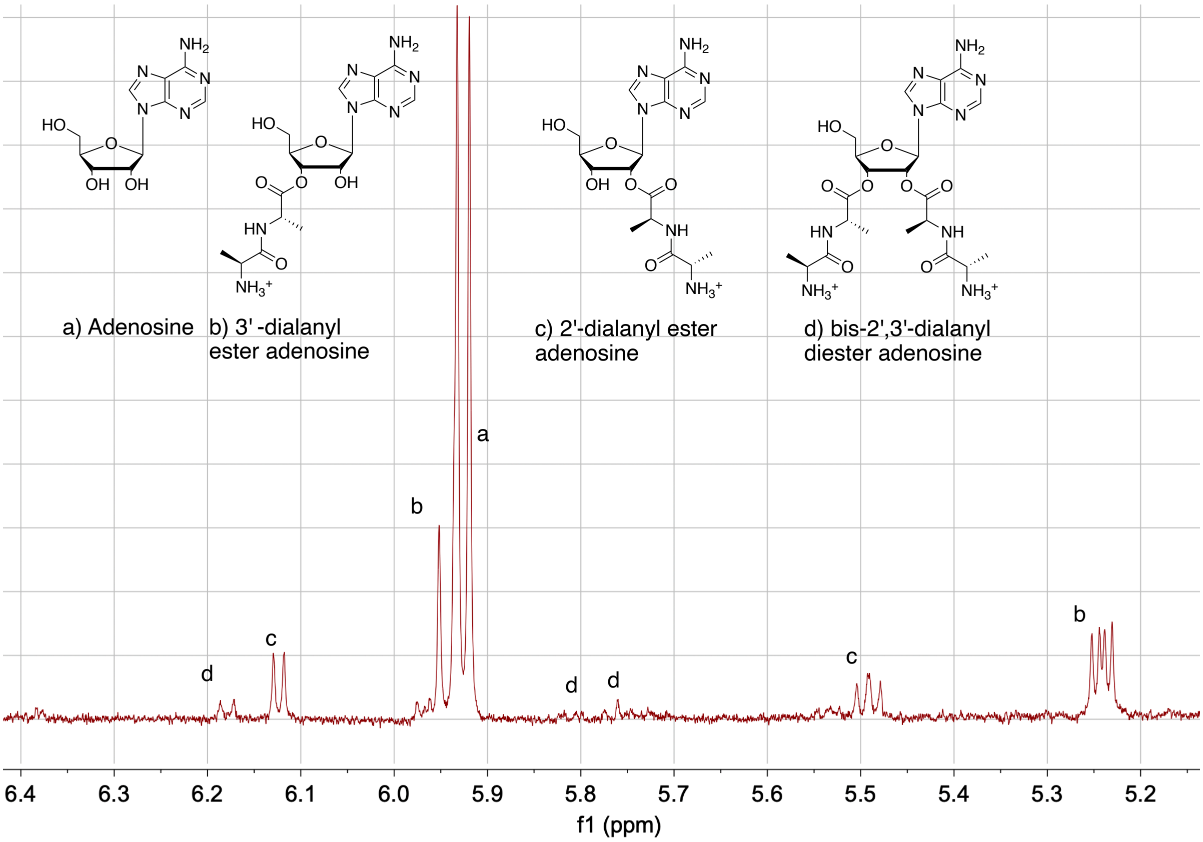

Figure S24. ^{1}H-NMR spectrum of synthetic standards of the 2'/3'-Ala-Ala-ester adenosines including adenosine and bis-2',3'-Ala-Ala-diester adenosine.

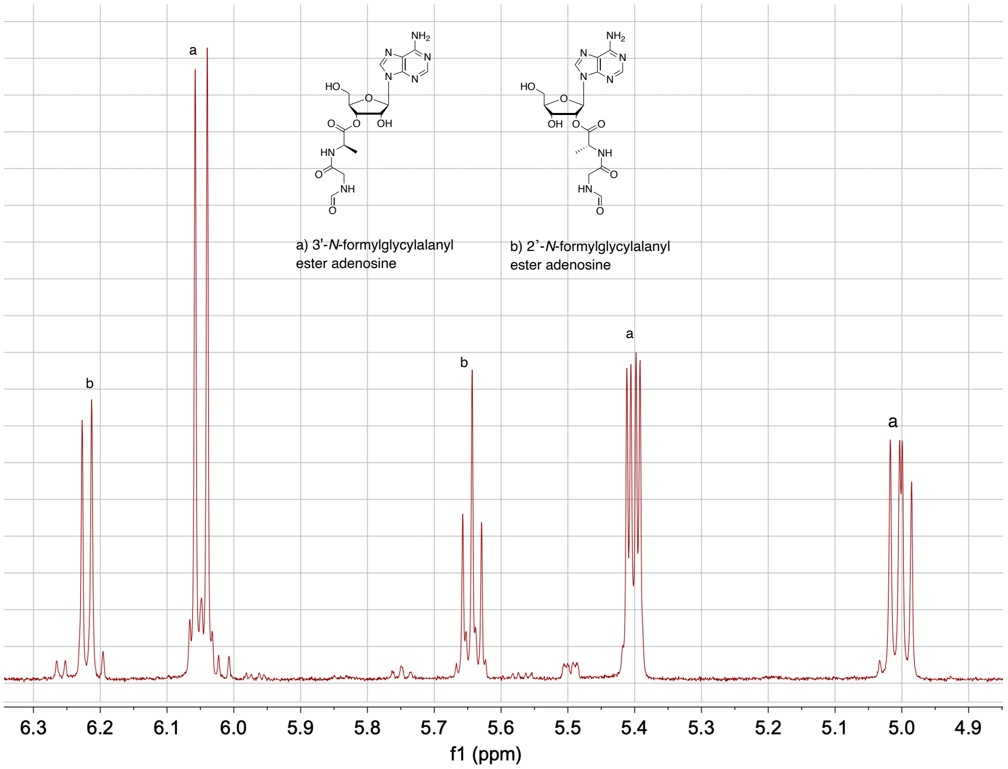

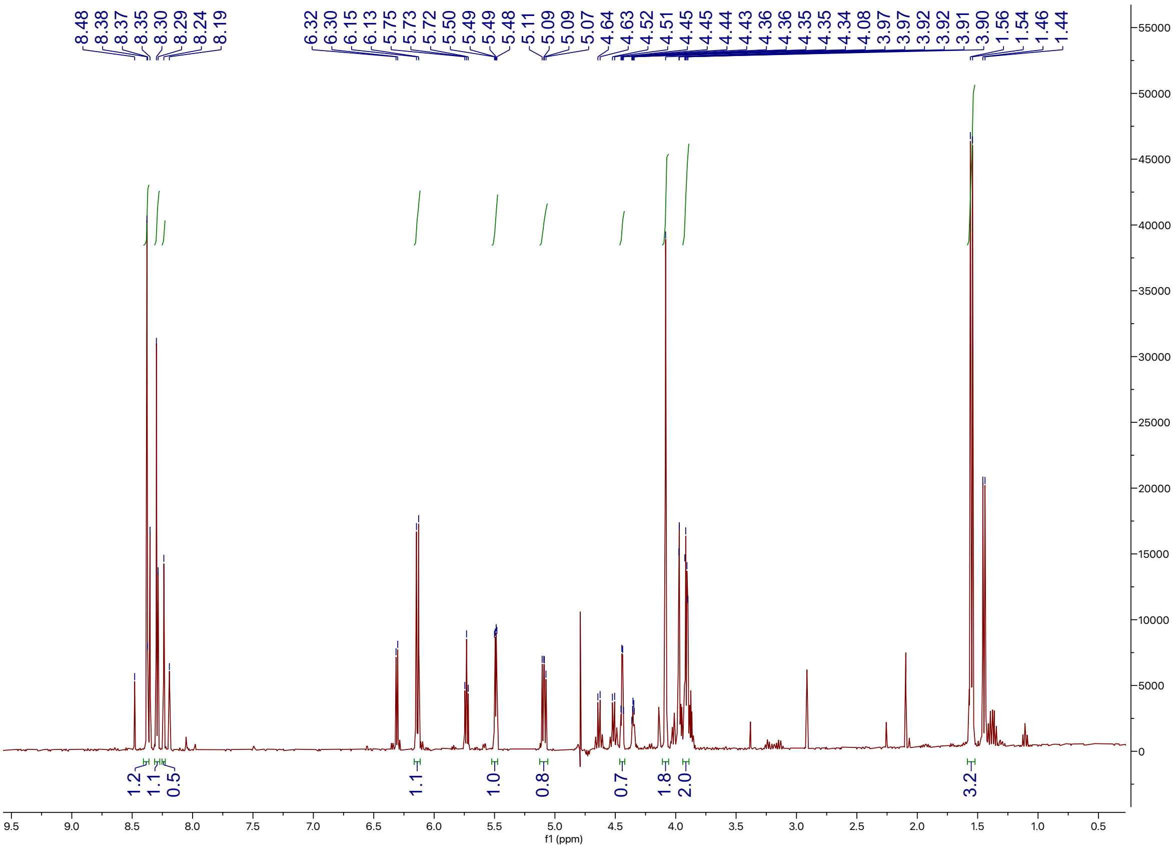

Figure S25. ^{1}H-NMR spectra of synthetic standards of the 2'/3'-fGly-Ala-ester adenosines.

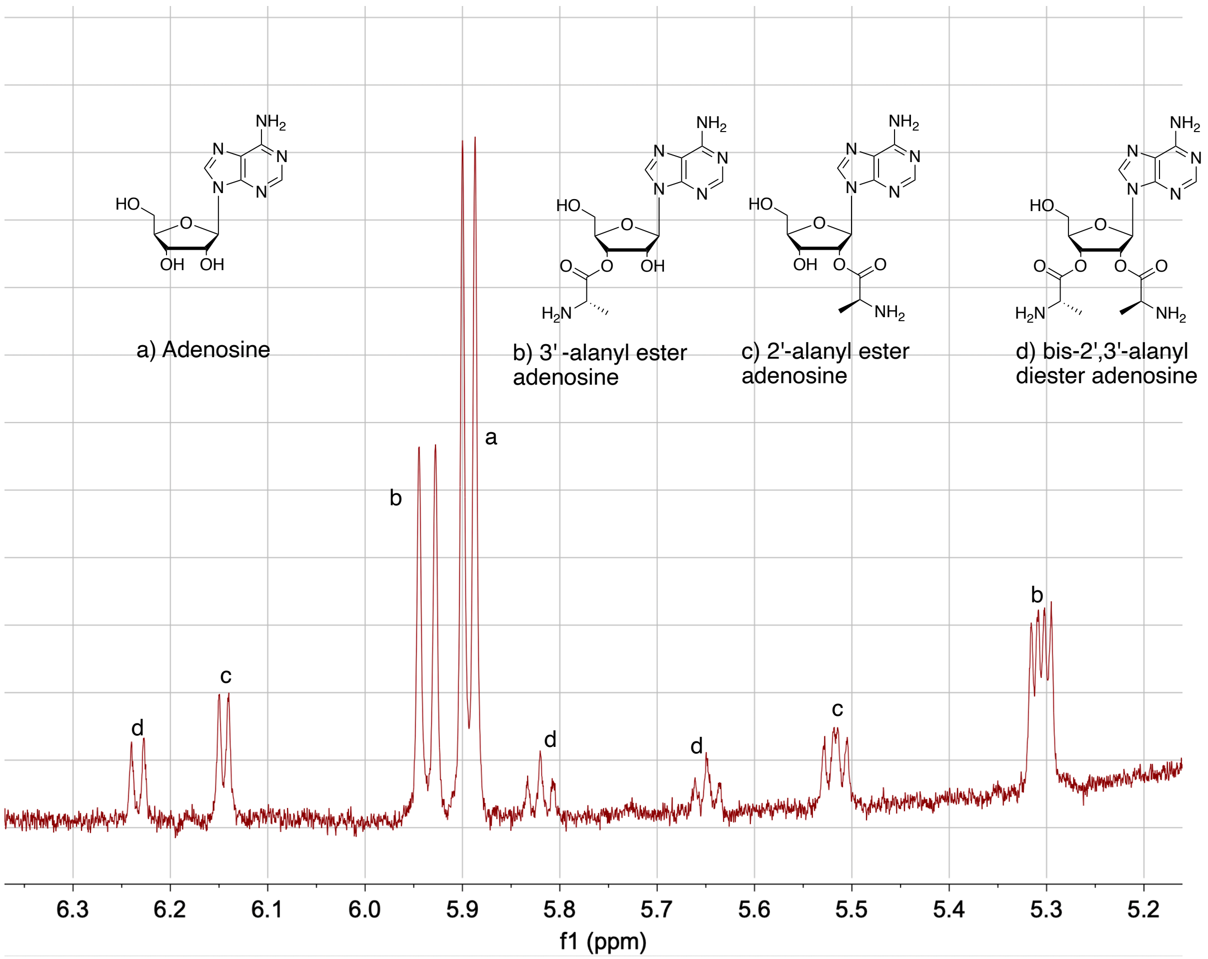

Figure S26. ^{1}H-NMR spectrum of synthetic standards of the 2'/3'-Ala-ester adenosines including adenosine and bis-2',3'-Ala-Ala-diester adenosine.

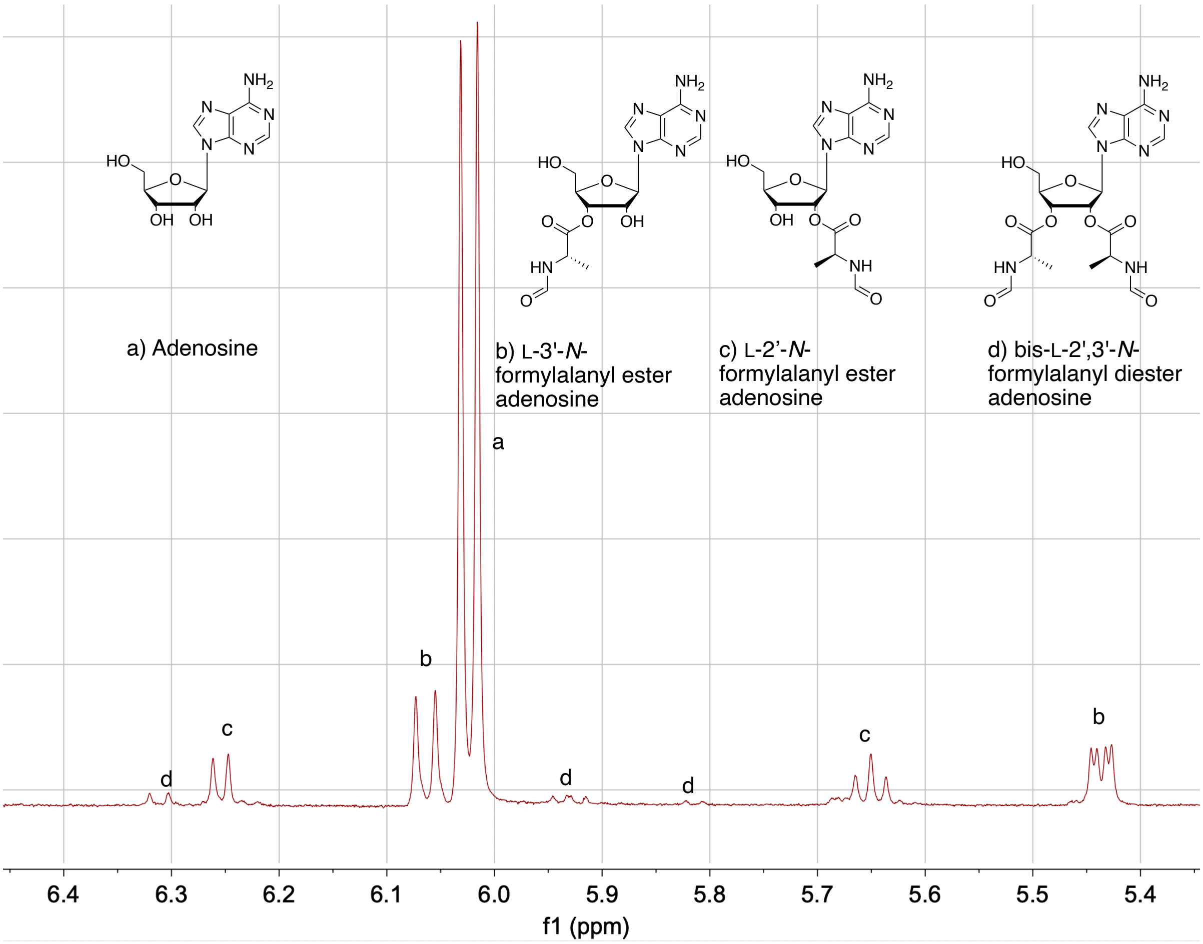

Figure S27. ^{1}H-NMR spectrum of synthetic standards of the L-2'/3'-*N*-fAla-ester adenosines including adenosine and bis-L-2',3'-*N*-fAla,fAla-diester adenosine.

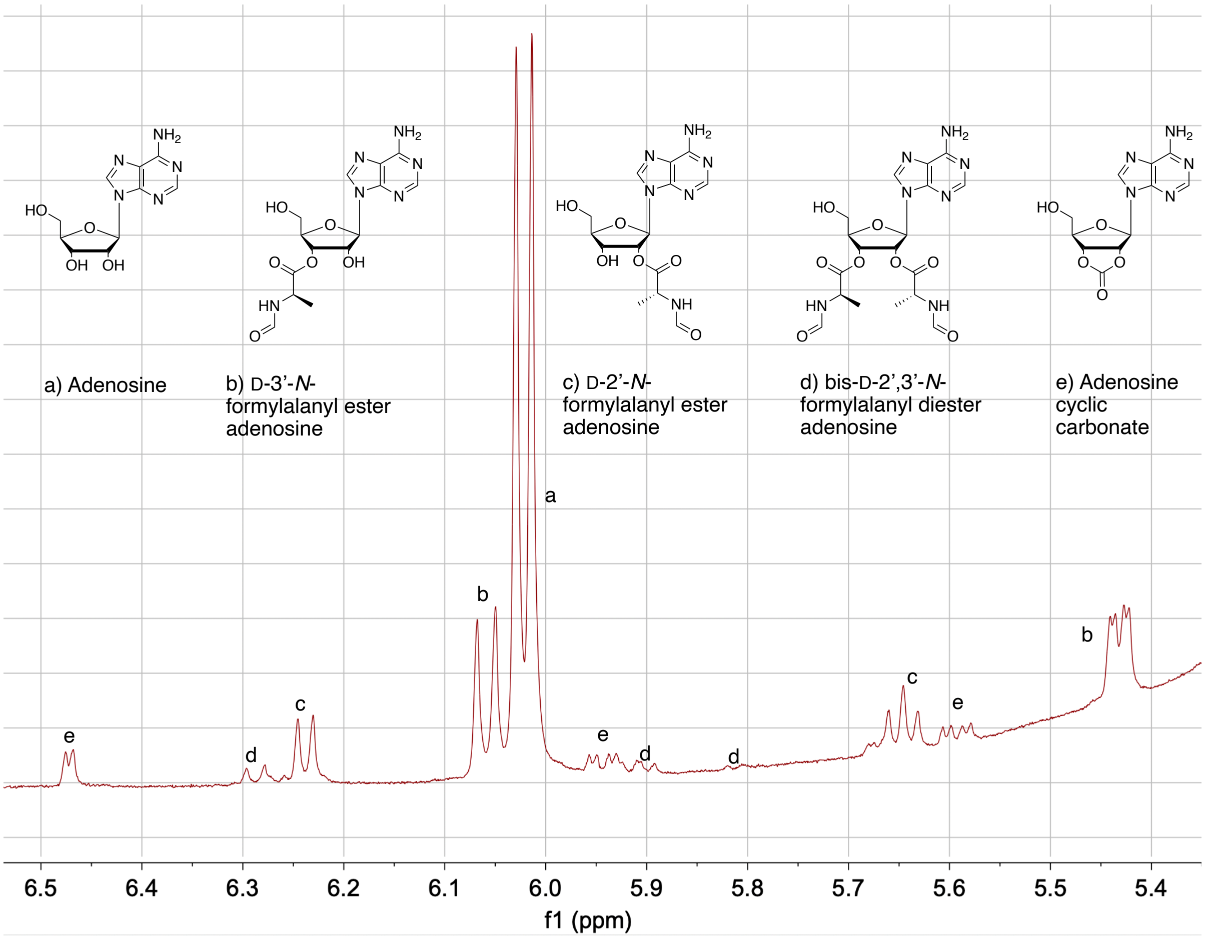

Figure S28. ^{1}H-NMR spectrum of synthetic standards of the D-2'/3'-*N*-fAla-ester adenosines including adenosine, bis-D-2',3'-*N*-fAla,fAla-diester adenosine and adenosine cylic-carbonate.

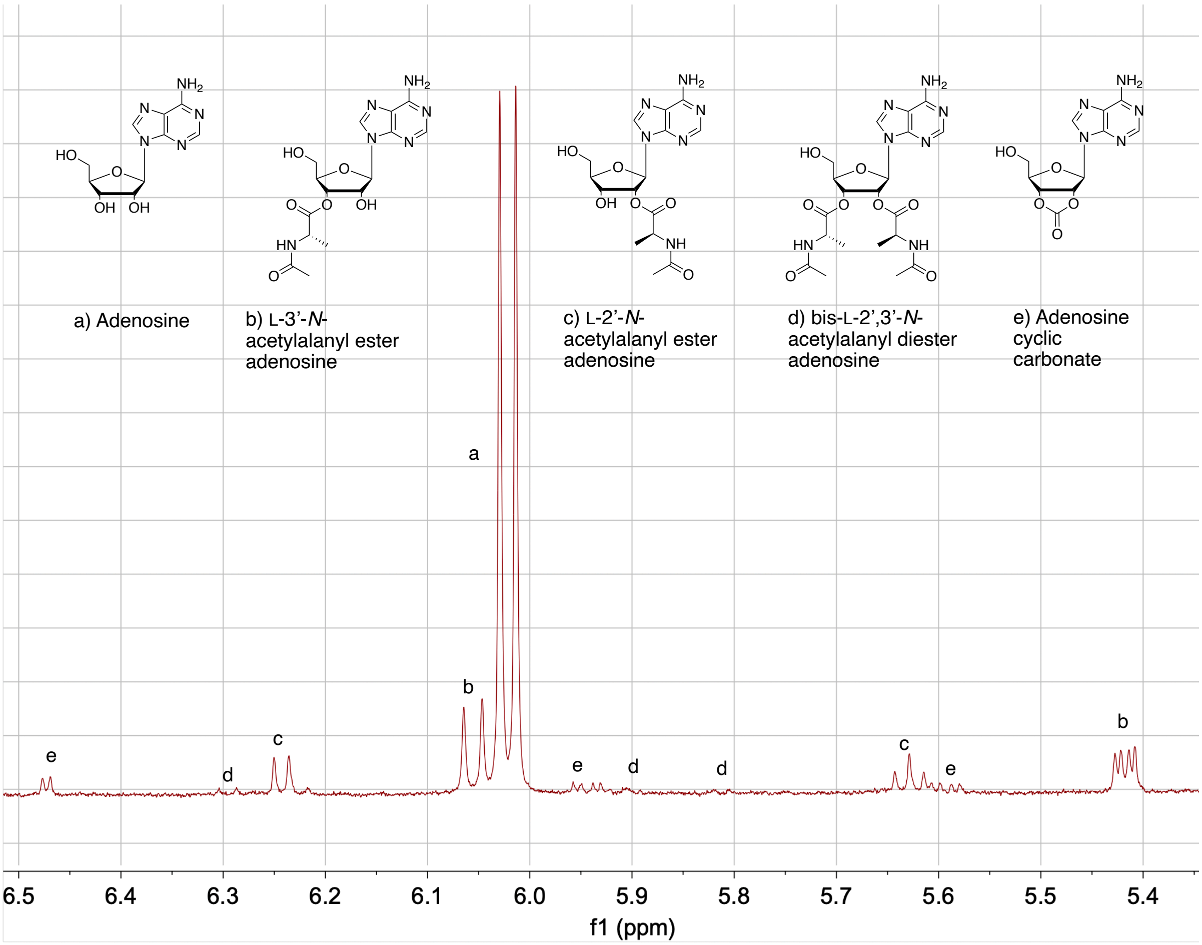

Figure S29. ^{1}H-NMR spectrum of synthetic standards of the L-2'/3'-*N*-AcAla-ester adenosines including adenosine, bis-L-2',3'-*N*-AcAla,AcAla-diester adenosine and adenosine cyclic carbonate.

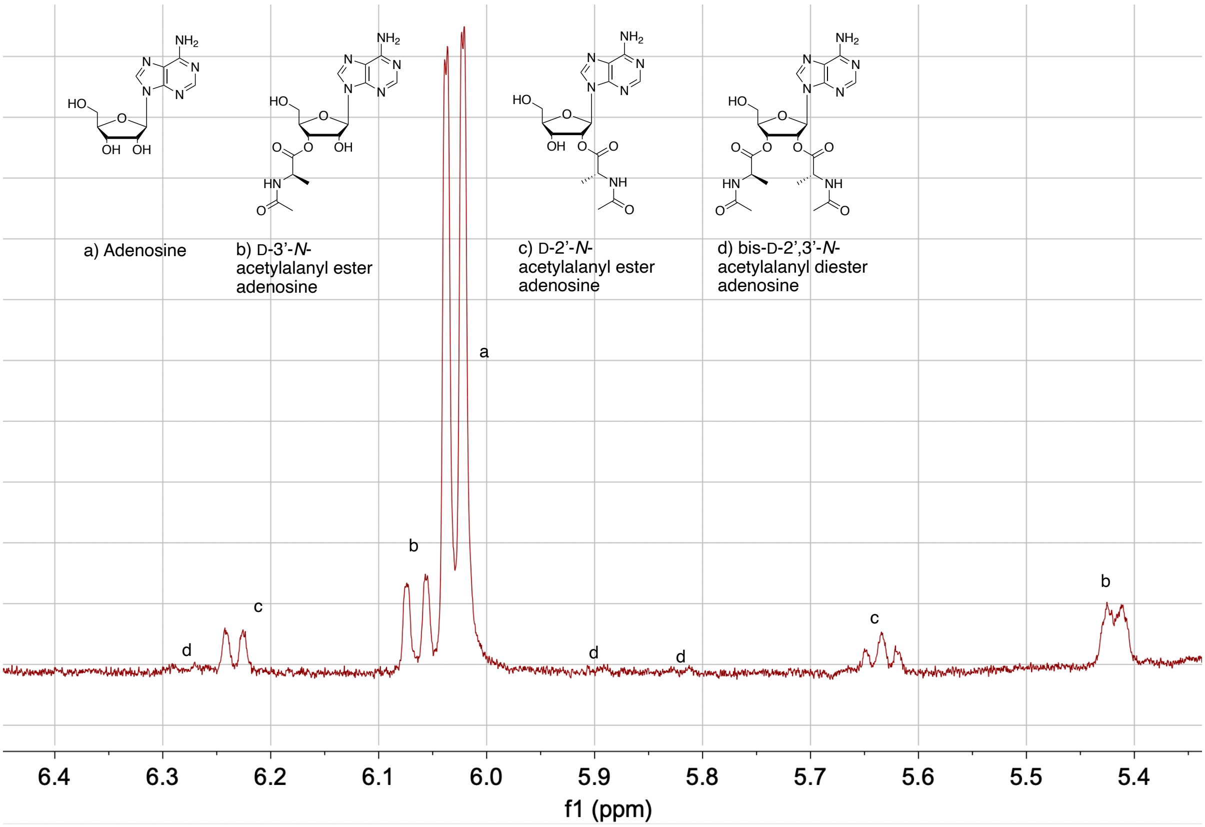

Figure S30. ^{1}H-NMR spectrum of synthetic standards of the D-2'/3'-*N*-AcAla-ester adenosines including adenosine and bis-D-2',3'-*N*-AcAla,AcAla-diester adenosine.
